# SCMR Position Paper (2020) on clinical indications for cardiovascular magnetic resonance

**DOI:** 10.1186/s12968-020-00682-4

**Published:** 2020-11-09

**Authors:** Tim Leiner, Jan Bogaert, Matthias G. Friedrich, Raad Mohiaddin, Vivek Muthurangu, Saul Myerson, Andrew J. Powell, Subha V. Raman, Dudley J. Pennell

**Affiliations:** 1 Department of Radiology, E.01.132, Utrecht University Medical Center, Heidelberglaan 100, 3584CX Utrecht, The Netherlands; 2 Department of Radiology, University Hospitals Leuven, Leuven, Belgium; 3 Department of Imaging and Pathology, Catholic University Leuven, Herestraat 49, 3000 Leuven, Belgium; 4 Departments of Medicine and Diagnostic Radiology, McGill University, 1001 Decarie Blvd., Montreal, QC H4A 3J1 Canada; 5 Department of Radiology, Royal Brompton Hospital, Sydney Street, Chelsea, London, SW3 6NP UK; 6 National Heart and Lung Institute, Imperial College, South Kensington Campus, London, SW7 2AZ UK; 7 Centre for Cardiovascular Imaging, Science & Great Ormond Street Hospital for Children, UCL Institute of Cardiovascular, Great Ormond Street, London, WC1N 3JH UK; 8 Division of Cardiovascular Medicine, Radcliffe Department of Medicine, Oxford Centre for Clinical Magnetic Resonance Research (OCMR), University of Oxford, John Radcliffe Hospital, Oxford, OX3 9DU UK; 9 Department of Cardiology, Boston Children’s Hospital, 300 Longwood Avenue, Farley, 2nd Floor, Boston, MA 02115 USA; 10 Department of Pediatrics, Harvard Medical School, 300 Longwood Avenue, Farley, 2nd Floor, Boston, MA 02115 USA; 11 Krannert Institute of Cardiology, Indiana University School of Medicine, 340 West 10th Street, Fairbanks Hall, Suite 6200, Indianapolis, IN 46202-3082 USA; 12 Royal Brompton Hospital, Sydney Street, Chelsea, London, SW3 6NP UK; 13 Imperial College, South Kensington Campus, London, SW7 2AZ UK

## Abstract

The Society for Cardiovascular Magnetic Resonance (SCMR) last published its comprehensive expert panel report of clinical indications for CMR in 2004. This new Consensus Panel report brings those indications up to date for 2020 and includes the very substantial increase in scanning techniques, clinical applicability and adoption of CMR worldwide. We have used a nearly identical grading system for indications as in 2004 to ensure comparability with the previous report but have added the presence of randomized controlled trials as evidence for level 1 indications. In addition to the text, tables of the consensus indication levels are included for rapid assimilation and illustrative figures of some key techniques are provided.

## Introduction

Cardiovascular magnetic resonance (CMR) is established in clinical practice for the diagnosis and management of diseases of the cardiovascular system. However, current guidelines for when this technique should be employed in clinical practice have not been revised since the previous Consensus Panel report of 2004 [1]. Considerable technical and practice advances have been made in the intervening years and the level of interest from clinicians in this field is at an unprecedented level. This Consensus Panel report updates these guidelines. As CMR is a multidisciplinary technique with international interest, the Consensus Panel was composed of European and American cardiologists and radiologists, all of whom are internationally recognized experts in the field of CMR, actively practice CMR and have played important roles in its development and clinical application. The coordinating authors (Leiner and Pennell) assembled the list of experts at the request of the Society for Cardiovascular Magnetic Resonance (SCMR) Executive Committee, ensuring coverage of all classes of indications. This list was subsequently approved by the SCMR Executive Committee as well as the Board of Trustees prior to the start of the writing process. The Consensus Panel was originated, approved and funded in its activities by the SCMR.

The Consensus Panel recommendations are based on evidence compiled from the literature and expert experience. Consensus on the evidence classes was achieved by a modified Delphi process. First the section (co-)authors suggested evidence classes for the indications in their respective sections. These were reviewed by all other authors in at least three written rounds of commentary. Evidence classes were finalized with all authors of the documents present in a telephone conference. When insufficient evidence is present in the literature, this is indicated in the report and recommendations are made conservatively under these circumstances. The appropriateness of using CMR is described for the frequent disease entities where imaging information may be warranted. The diagnostic use of CMR will be described in the context of other, competing non-invasive imaging techniques, with particular emphasis on the differential indications with respect to echocardiography.

The usefulness of CMR in specific diseases is summarized by means of the following classification:

- Class I = provides clinically relevant information and is usually appropriate; may be used as first line imaging technique; usually supported by substantial literature or randomised controlled trial(s).
- Class II = provides clinically relevant information and is frequently useful; other techniques may provide similar information; supported by limited literature.
- Class III = provides clinically relevant information but is infrequently used because information from other imaging techniques is usually adequate.
- Class Inv = potentially useful, but still investigational.

This classification is not meant to equate to American Heart Association/American (AHA), American College of Cardiology (ACC) and European Society of Cardiology (ESC) consensus documents. We have used nearly the identical classification system that was used for the original Consensus Panel report of 2004, in order to maintain parity with that report so that advances in the field can be readily identified. Because of broad variation in the number of relevant papers between topics, we did not use an arbitrary number of papers for each class. Class 1 indications required the highest level of evidence, for example affirmative randomized controlled trials (RCT) results for use of perfusion CMR. The categories were populated by consensus. The quality of the publications (impact factor, RCT or not, etc.) was germane to the Consensus Panel discussion of ranking of class throughout the discussions. Thus, the only change from 2004 is to add the positive outcome of RCTs in CMR as evidence for level 1 indications. It should also be noted that the classification system for imaging technologies does not easily accord with that of therapeutic trials because the datasets are smaller, multicentre trials are uncommon and randomized controlled trials the exception. The continuing technical and clinical advances in CMR will change the indication’s tables, and therefore between formal reports, the Consensus Panel may post updates online.

## Congenital heart disease

CMR is a well-established modality for the diagnosis and follow-up of patients with congenital heart disease (CHD) (Table 1). This is because it provides unrestricted evaluation of the intracardiac and vascular anatomy, and reference standard measurements of the ventricles and blood flow. In general, transthoracic echocardiography (TTE) continues to be used as the first-line method of assessing cardiovascular anatomy and function in patients with CHD. However, when acoustic window limitations preclude an adequate assessment or more reliable quantification of ventricular parameters and blood flow is required, CMR is often indicated. In addition, the tissue characterization capabilities of CMR (e.g. fibrosis detection) can have a significant impact on patient management [2–6]. CMR has also been used to replace invasive diagnostic catheterization for assessment of CHD as it provides comparable anatomic information without exposure to ionizing radiation or the risks of an invasive procedure [7, 8]. CMR’s advantages over computed tomography (CT) include again the avoidance of ionizing radiation along with superior ventricular function assessment, blood flow measurement, and tissue characterization.

**Table 1:** Indications for CMR in congenital heart disease

Indication	Class
1. General	
Initial evaluation and follow-up of congenital heart disease	C II/A I
Evaluation of right and left ventricular volumes, mass, and function	I
Measurement of the pulmonary-to-systemic flow ratio	I
2. Shunts lesions	
Patent foramen ovale	III
Atrial septal defects	C III/A II
Sinus venosus defects	I
Anomalous pulmonary venous connection	I
Ventricular septal defects	C III/A II
Atrioventricular septal defects	C III/A II
Patent ductus arteriosus	C III/A II
Aorto-pulmonary window	C III/A II
Systemic-to-pulmonary artery collaterals	I
3. Valve lesions^a^	
Tricuspid valve disease, including Ebstein disease	II
Pulmonary valve disease	II
Mitral valve disease	III
Aortic valve disease	II
4. Arterial lesions	
Aortic coarctation and interrupted aortic arch	C II/A I
Vascular rings	I
Supravalvular aortic stenosis	C III/A II
Coronary anomalies	C II/A I
Pulmonary artery stenosis	C II/A I
5. Conotruncal lesions	
Tetralogy of Fallot	C II/A I
Double outlet right ventricle	I
D-loop transposition of the great arteries	C II/A I
Congenitally corrected transposition of the great arteries	I
Truncus arteriosus	I
6. Complex disease	
Heterotaxy syndrome	I
Single ventricle heart disease	I

C: children; A: adult

^a^For non-congenital valve lesions in adults, please refer to Table 7

When performing CMR in CHD patients, it is important to tailor the acquisition parameters to the faster heart rates and smaller structures in children, as well as the limited breath-holding ability of children and poorly compensated adult CHD patients. It is also necessary to have thorough knowledge of CHD anatomy and the extensive array of surgical and catheter interventions. Therefore, it is highly recommended that CMR in CHD patients is performed in centers with sufficient experience and expertise.

### General

In younger children with CHD, TTE is often sufficient for initial diagnosis. However, CMR can provide important additional information in lesions with complex intracardiac anatomy or vascular abnormalities. In adults with CHD, TTE is often limited by poor image quality and reduced field of view. Thus, CMR with its reliable visualization of intracardiac and vascular anatomy takes on a central role in the routine non-invasive imaging of these patients. This indication has been recognized in recent guidelines regarding multi-modality imaging and the management of adults with CHD [9–11]. Another key indication for CMR in CHD is the assessment of ventricular volumes, mass, and function. The clear delineation of endocardial and epicardial borders, and ability to quantify without geometric assumptions have established CMR as the clinical reference standard [12–14]. The advantages of CMR are particularly valuable for right ventricular (RV) assessment as its retrosternal position and complex shape make it difficult to reliably assess by TTE. This capability is especially relevant in CHD as many lesions are associated with a RV pressure or volume load. Several studies have demonstrated good reproducibility of RV measurements by CMR in CHD underscoring its suitability for serial assessment [15–18]. A final important general indication for CMR is assessment of blood flow. Multiple studies have shown that it provides accurate measurement of cardiac output and the pulmonary-to-systemic flow (Qp:Qs) ratio compared to invasive techniques [19–21].

### Shunt lesions

**Fig. 1:**
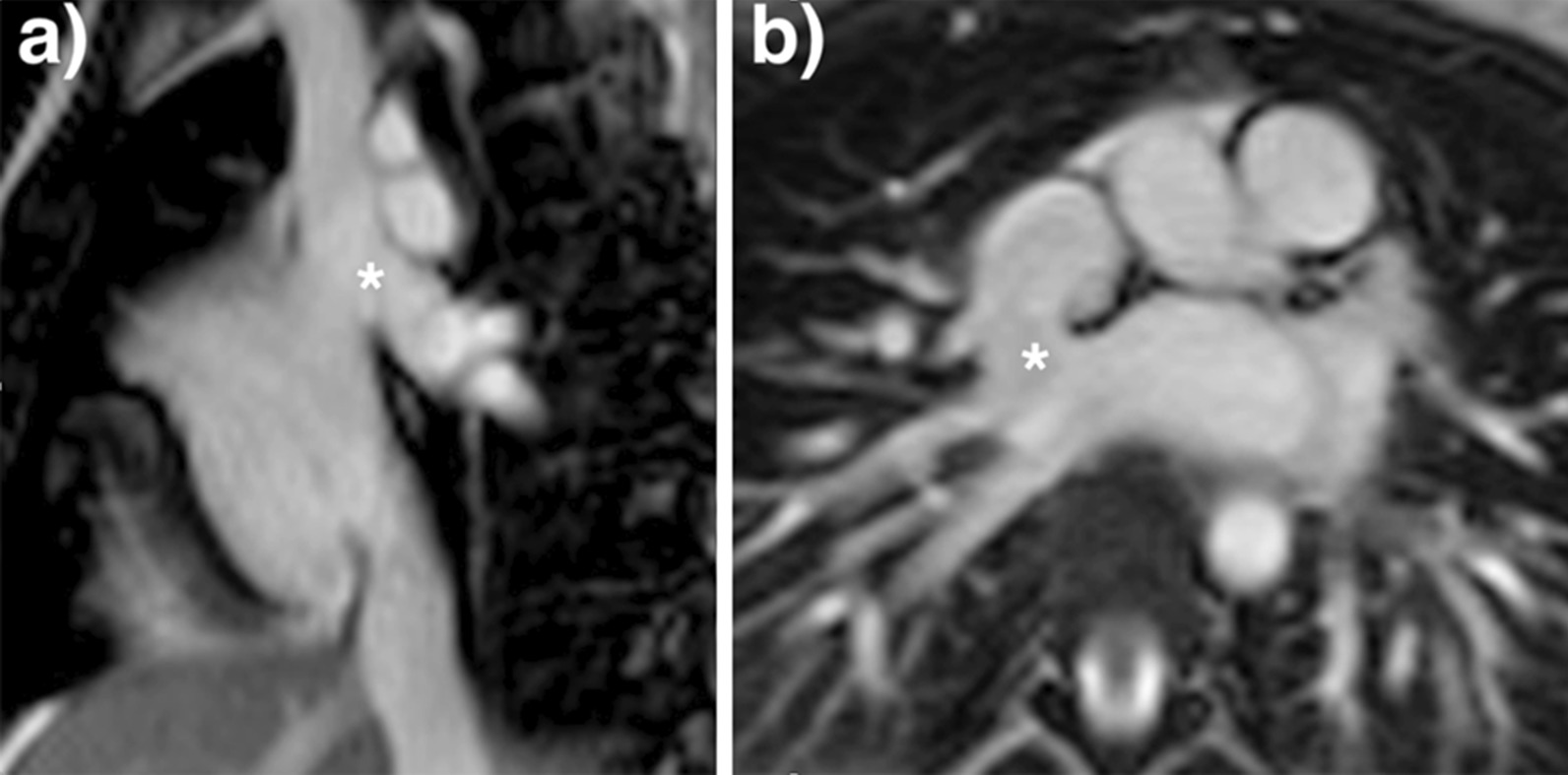
Balanced steady state free precession (bSSFP) cine images through a superior sinus venosus defect (*): **a** sagittal oblique view, **b** transverse oblique also showing anomalously draining right pulmonary veins

For shunt lesions, CMR is used to delineate anatomy and determine physiological importance through measurement of the Qp:Qs ratio and ventricular volumes. This is vital information for determining the necessity and timing of surgical or catheter interventions. Studies have shown that CMR can provide definitive diagnosis and evaluation of sinus venosus defects and anomalous pulmonary venous connection [22, 23] (Fig. 1). This is relevant as these lesions are difficult to image with TTE, particularly in older patients. Several studies have also shown that CMR provides a comprehensive evaluation of the morphology and physiological importance of secundum atrial septal defects, and can determine candidacy for transcatheter or surgical closure [24–26]. CMR may be especially useful in adult patients who have findings (e.g., murmur or RV dilation) that raise a suspicion for an atrial septal defect or other shunt lesion that is not definitively resolved by TTE. Here, CMR provides a non-invasive alternative to transesophageal echocardiography (TEE) with better sensitivity for anomalous pulmonary venous connections. Patent foramen ovale is usually diagnosed with echocardiography though there are CMR techniques to address this question [27, 28]. Ventricular septal defects are generally diagnosed and managed using echocardiography. However, CMR with 3D imaging may be useful for delineation of complex or multiple defects [29]. In addition, CMR measurement of the Qp:Qs ratio and left ventricular (LV) volume may be important in determining management in older children and adults [9]. Most patients with atrioventricular septal defects are diagnosed in childhood by TTE and undergo surgical repair. Subsequently, atrioventricular valve regurgitation may develop and CMR provides a reliable quantitative assessment of its severity [30, 31] and insight into its mechanism [32]. Patent ductus arteriosus and aorto-pulmonary window typically present in infancy where echocardiography is usually adequate for diagnosis and management. When this evaluation is insufficient or the patient is older, CMR techniques can be used to define the vascular anatomy and assess the magnitude of the shunt. In patients with systemic-to-pulmonary artery collaterals, CMR can be used to quantify the amount of collateral flow [33, 34] and identify larger vessels that may be suitable for catheter interventions.

### Valve lesions

CHD may be associated with abnormalities in the cardiac valves. Echocardiography is often the primary modality for assessing valve morphology and function. CMR is principally used to evaluate the physiological impact of valve regurgitation by measuring the regurgitation volume, regurgitant fraction, and ventricular size and function. This information plays an important role in deciding the timing of mechanical interventions. With valve stenosis, CMR can be used to define orifice size [35] but may underestimate the peak velocity and estimated pressure gradient [36]. In unrepaired Ebstein’s anomaly, CMR assessment of the abnormal tricuspid valve leaflets, tricuspid regurgitation, and RV volumes and function can be used to determine the suitability for operative repair [37, 38] and predict major adverse cardiac events and atrial arrhythmia [39]. Following repair, CMR can demonstrate the extent of ventricular remodeling [40, 41].

### Arterial lesions

**Fig. 2:**
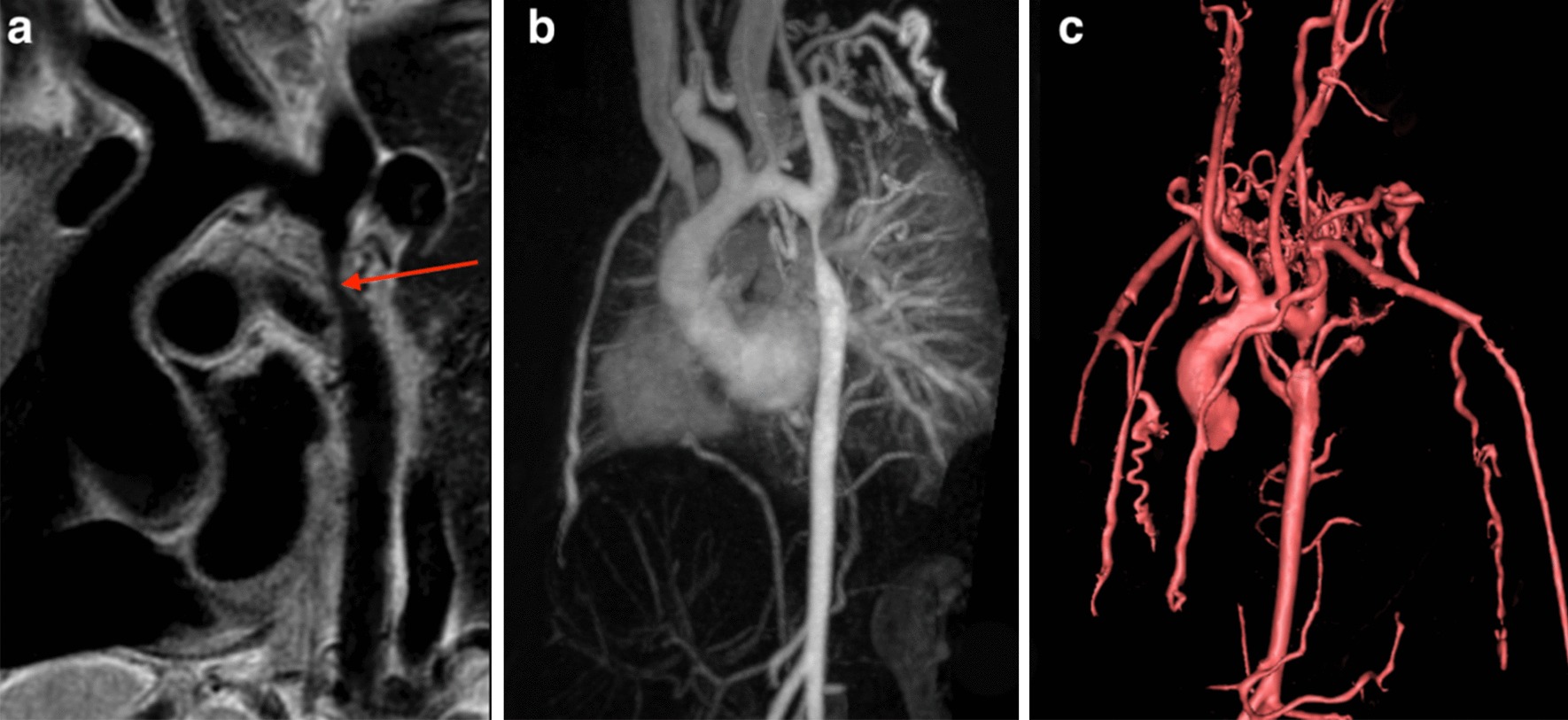
Multiple CMR methods of imaging coarctation of the aorta: **a** sagittal oblique 2D black blood, **b** maximal intensity projection gadolinium enhanced CMR angiogram (CMRA) and **c** volume rendering of gadolinium enhanced CMRA

Congenital abnormalities of the aorta, pulmonary arteries, and coronary arteries may occur in isolation or in association with other CHD lesions. The ability to assess these structures by echocardiography becomes progressively more difficult as patients become larger, and CMR angiography techniques serve as an important non-invasive alternative [42–44]. In coarctation of the aorta, CMR is used for assessment before and after intervention (Fig. 2). In addition to delineating the anatomy [45], CMR provides information on severity through assessment of the peak velocity at the obstruction site, extent of collateral flow, and ventricular hypertrophy [46–51]. In adults, the combination of clinical assessment and CMR has been shown to provide a better “cost-effective” yield compared with a combination that relies on echocardiography as the primary imaging modality [52]. Vascular rings can be fully delineated using CMR, including the trachea and main bronchi to identify associated compression [53, 54]. CMR reliably depicts congenital abnormalities in the course of the proximal coronary arteries [55, 56], and diagnosis has been shown to predict major adverse cardiac events [57]. Compared to X-ray angiography, CMR better demonstrates the coronary course with respect to the semilunar valves [58], which is crucial for risk-stratification and surgical planning. CMR provides an accurate assessment of main and branch pulmonary artery dimensions [44]. In the setting of branch pulmonary artery stenosis, it can quantify differential lung perfusion [59, 60], which has been shown to better predict symptoms than anatomic measurements [61]. Lastly, CMR has been used to diagnose supravalvar aortic stenosis and characterize its physiological importance [62].

### Conotruncal lesions

Conotruncal lesions include tetralogy of Fallot, transposition of the great arteries (TGA), double outlet RV, and truncus arteriosus, among others. The primary imaging modality for these conditions in younger children is echocardiography. CMR may augment the preoperative assessment of double outlet RV by better characterizing the intracardiac anatomy, particularly through use of virtual and 3D printed models [29, 63]. CMR can also be used define the pulmonary blood supply including aorto-pulmonary collaterals in patients with complex pulmonary stenosis or atresia [8]. In older children and adults with repaired conotruncal lesions, CMR assumes an important role as the clinical concerns often involve the pulmonary arteries, aorta, coronary arteries, pulmonary valve, intracardiac baffles, and RV, all relative strengths compared to echocardiography. In patients with repaired tetralogy of Fallot, CMR-derived parameters inform risk-stratification [3, 64–66] and referral for pulmonary valve replacement [67–69], and figure prominently in published clinical management guidelines [9–11, 70]. In patients with d-loop TGA who have undergone an atrial switch procedure, CMR is used to evaluate the atrial baffles, and systemic RV volumes, function [18], and scar [2]; abnormal findings have been shown to predict outcome [71]. For d-loop TGA patients who have had an arterial switch operation, CMR indications include monitoring for pulmonary artery stenosis, semilunar valve regurgitation, and coronary artery obstruction [72–74]. Congenitally-corrected TGA patients require CMR for surveillance of their systemic RV; those who are post-operative need assessment of intracardiac baffles, arterial conduits, coronary stenosis, and ventricular function [75, 76]. The surgical repair of patients with truncus arteriosus as well as other conotruncal lesions may include the placement of a ventricle-to-pulmonary artery conduit and these are prone to develop stenosis and regurgitation over time. TTE imaging is often difficult due to the retrosternal position of the conduit while CMR provides accurate anatomic and functional assessment [75–78].

### Complex lesions

The reliable visualization of intracardiac and vascular anatomy, large field of view, and 3D imaging capabilities all make CMR well-suited for the diagnosis of complex cardiovascular anatomic arrangements, such as those seen in more severe forms of heterotaxy syndrome [79, 80]. CMR is a non-invasive alternative to invasive cardiac catheterization and often provides a more comprehensive evaluation than echocardiography. CMR also has an important role in the diagnostic evaluation and serial follow-up of patients with single ventricle heart disease. TTE is the primary imaging tool during the initial evaluation since most patients present in the newborn period and their acoustic windows are typically adequate; in some cases, CMR may be used to determine whether a one versus two-ventricle repair should be pursued [81, 82]. Studies have shown that later in their course, CMR outperforms TTE and can substitute for routine diagnostic catheterization in selected patients prior to the bidirectional Glenn shunt or a hemi-Fontan procedure (second-stage palliation) [7, 83–85], and prior to the Fontan procedure (third-stage palliation) [86–88]. Moreover, CMR measurements of systemic-to-pulmonary artery collateral flow predict post-operative outcomes such as hospital length of stay [89–91]. Following the Fontan procedure, patients remain at risk for numerous complications including ventricular and valve dysfunction, Fontan baffle obstruction, pulmonary artery stenosis, aortic coarctation, systemic-to-pulmonary venous collateral formation, and intracardiac thrombus formation. CMR has a key role in surveillance for these complications as the evaluation by TTE alone is often incomplete [92, 93]. Finally, CMR-derived parameters such as ventricular volume and myocardial fibrosis have been shown to be associated with adverse outcomes [5, 94].

## Acquired vascular disease

CMR is a highly reliable modality for depiction of the presence and extent of acquired vascular disease in virtually all of the large and medium sized arteries in the body (Table 2). A variety of techniques exist to depict both the vascular lumen as well as the vessel wall. The current standard of reference is contrast-enhanced (CE) CMRA. CE-CMRA is obtained during first arterial passage of an intravenous bolus injection of gadolinium-based contrast agents (GBCA). On modern CMR scanners, virtually all CE-CMRA applications can be performed with a single dose of contrast agent (0.1 mmol/kg). If the area of interest exceeds a single field-of-view (FOV), as is the case for total body or lower extremity artery imaging, a moving table protocol can be used whereby the contrast bolus is followed over multiple FOV by rapid table movement. Typical spatial resolution is in the order of 1.0 × 1.0 (in-plane) × 1.0–2.0 (slice thickness) mm^3^. On modern hardware this high spatial resolution can be obtained in a single breath hold, which is necessary for chest and abdominal CE-CMRA. For some clinical indications it is advisable to use a multi-phase dynamic acquisition as this can provide information about the direction of blood flow as well as information about tissue perfusion. Since dynamic CE-CMRA demands a higher temporal resolution this typically comes at the expense of spatial resolution. In selected cases it can therefore be useful to perform both acquisitions with two separate injections of contrast agent. Although the vast majority of CE-CMRA studies are performed with GBCA, a substantial body of literature has been published on the use of ferumoxytol as a CMRA contrast agent. Ferumoxytol is an intravenous iron preparation for treatment of the anemia of chronic kidney disease [95]. It is a carbohydrate-coated, superparamagnetic iron oxide nanoparticle that leads to prolonged intravascular enhancement when patients are imaged with CE-CMRA sequences. Ferumoxytol-enhanced CE-CMRA can be obtained with much higher spatial resolution due to the much longer intravascular half-life of this contrast agent [96]. This pharmacokinetic profile can be advantageous when both high spatial resolution as well as cardiac and/or respiratory synchronization is desired, or in patients with contra-indications for administration of GBCA.

**Table 2:** Indications for CMR in acquired diseases of the vasculature

Indication	Class
1. Diagnosis and follow-up of thoracic aortic aneurysm including connective tissue diseases	I
2. Diagnosis and planning of stent treatment for abdominal aortic aneurysm	II
3. Follow-up of stented abdominal aortic aneurysm	III
4. Aortic dissection	
Diagnosis of acute aortic dissection	II
Diagnosis and follow-up of chronic aortic dissection	I
5. Diagnosis of aortic intramural hematoma	I
6. Diagnosis of penetrating aortic ulcers	I
7. Pulmonary artery anatomy and flow	I
8. Pulmonary emboli	
Diagnosis of central pulmonary emboli	III
Diagnosis of peripheral pulmonary emboli	III
Assessment of chronic pulmonary embolic disease	III
9. Assessment of aortic arch arteries	I
10. Assessment of aortic branch arteries including the Adamkiewicz artery	II
11. Assessment of carotid, vertebral and circle of Willis arteries	I
12. Assessment of upper extremity arteries	I
13. Assessment of hand arteries	II
14. Assessment of renal arteries	I
15. Assessment of mesenteric arteries	I
16. Assessment of pelvic and lower extremity arteries	I
17. Assessment of pulmonary veins	I
18. Assessment of thoracic, abdominal and pelvic veins	I
19. Assessment of lower extremity veins	I
20. Assessment of atherosclerotic plaque in the carotid artery	II
21. Assessment of atherosclerotic plaque in the aorta	II
22. Assessment of vascular wall inflammation in large and medium sized arteries	II
23. Assessment of aortic pulse wave velocity	Inv
24. Endothelial function	Inv

Over the past few years all major hardware vendors have also introduced non-contrast or native CMRA sequences on their platforms. These sequences utilize intrinsic contrast between flowing blood and stationary tissues as the basis to generate angiograms. Different technical approaches are also well described [97]. Major advantages of non-contrast CMRA include cost-savings and improved patient safety. They are also the optimal CMR technique for assessing aortic dissection as the dissection flap in better visualized than with CE-CMRA. Disadvantages include the slightly longer time needed for acquisition (several minutes compared to < 1 min for CE-CMRA), marginally lower spatial resolution, and reduced ability to provide 3D surface rendered images.

In addition to angiography, the wide variety of soft tissue contrast available on CMR (proton density, T1, T2, lipid-saturation) can be applied to vascular imaging to assess features of vessel wall such as haematoma/thrombus, inflammation, and atherosclerotic plaque. In addition to morphologic imaging of blood vessels, velocity mapping can be used to assess and measure the blood flow. Blood velocity and flow can be integrated across the cardiac cycle and the vessel lumen for reliable volume flow measurements. This information is complementary to the anatomical information obtained with the luminographic techniques and can add value in many clinical scenarios [98].

### Aorta

**Fig. 3:**
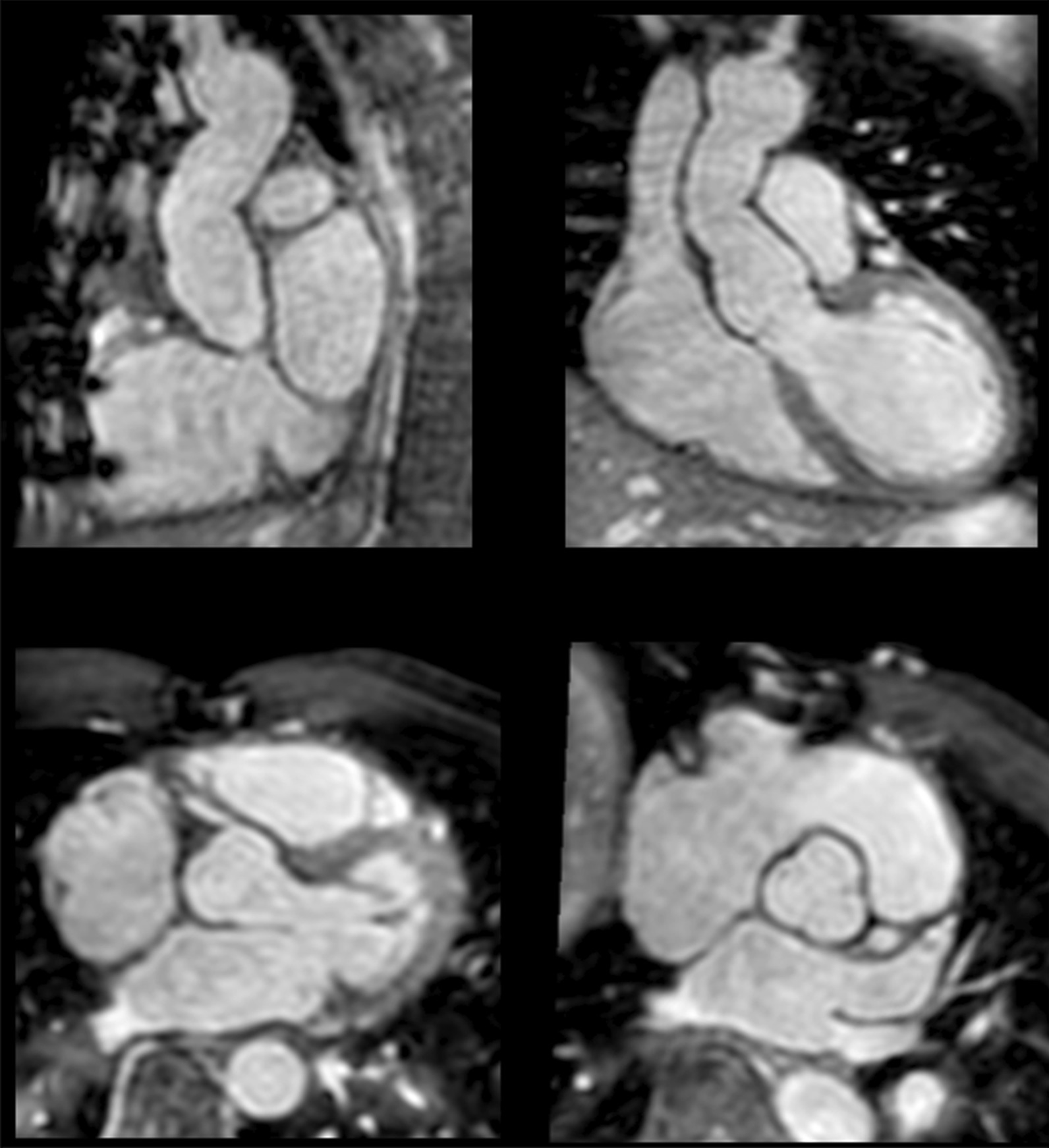
61-year old male followed up after ascending aorta replacment for Stanford type A ascending aortic dissection. Balanced steady state free precession (bSSFP) non-contrast CMRA of the ascending aorta synchronized to the diastolic rest period shows the aortic root and origin of the right coronary artery without motion artefacts. Note artefacts due to sternal wires

CMR techniques are well-suited to depict the thoracic and abdominal aorta. The high spatial resolution can be used for accurate assessment of aortic size, diameter and the presence and morphology of aortic aneurysms. The near isotropic spatial resolution ensures high quality multi-planar reformations (MPRs) which can be used to generate an accurate center-lumen line as well as depiction of side branches. Black blood techniques have been superseded by CE-CMRA and non-contrast CMRA techniques. The latter technique is particularly useful because image acquisition is typically synchronized to the diastolic rest period which greatly improves evaluation of the aortic root and leads to sharper aortic wall delineation because there is no blurring due to vessel pulsatility (Fig. 3). The advantage of CE-CMRA however is the ability to perform a multiphasic acquisition which can be used to depict contrast agent dynamics. Post-gadolinium T1-weighted CMR, especially with fat saturation, is helpful in identifying areas of peri-aortic inflammation in mycotic aneurysms or suspected vasculitis [99–101]. Inflammatory aortic aneurysms can have a thick rind of tissue encircling the anterior and lateral aspects of the aorta, which typically enhances with gadolinium [102]. Although not as widely used as CT for pre- and post-operative evaluation of aortic stent-grafts, CMR provides comparable information with regard to pre-stent anatomy but is more sensitive for detection of post-stent leaks for certain stent types [103–105]. A limitation of CMR used to be its inability to visualize calcium, which is important for stent graft planning. However, a recent study has shown the feasibility of a novel CMR sequence to accurately depict arterial calcifications with excellent agreement to CT angiography (CTA) [106].

Another important component of the aortic imaging protocol is measurement of flow. Although 2D flow sequences are still the most widely used technique, there is high interest protocols that allow determination of flow in a three-dimensional volume over the cardiac cycle (also known as ‘4D flow’). This can be particularly interesting in the chest since it enables simultaneous imaging of flow in the cardiac chambers as well as the large vessels in a single acquisition [107].

**Fig. 4:**
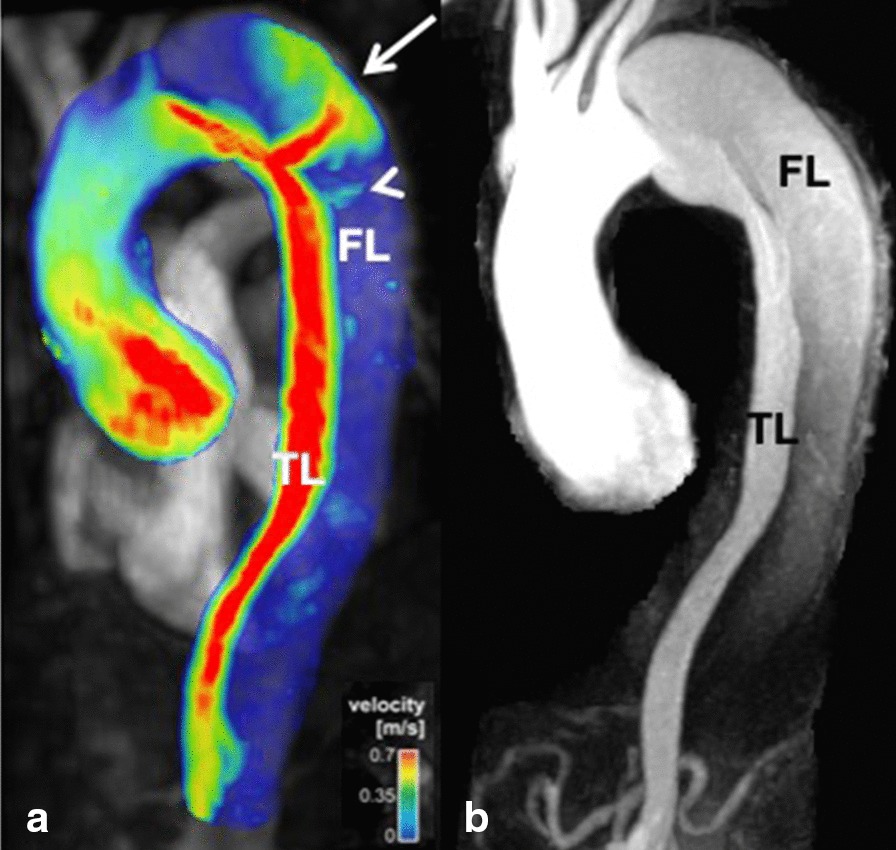
4D flow CMR velocity maximal intensity projection (MIP) (A) and time-resolved CMRA (TR-CMRA) MIP in a patient with type B aortic dissection. While TR-CMRA demonstrates dynamic filling of the proximal false lumen (FL), 4D flow exhibits a prominent entry tear jet impinging on the wall of the distal arch (white arrow). At least one smaller hemodynamically active fenestration is also seen more distal to the primary entry tear (arrowhead). TL: true lumen. Figure courtesy of Bradley D. Allen, MD MS, and Michael Markl, Ph.D., Northwestern University, Chicago, IL

Acute thoracic aortic syndromes include aortic dissection and limited intimal tear, intramural hematoma and penetrating aortic ulcer [108]. Aortic dissection remains a well-established indication for CMR although CT may be more widely available in the acute setting and dealing with acutely ill patients can be complicated in the CMR environment. A recent systematic review found that initial diagnostic evaluation with CMR had a sensitivity of 95–100% and a specificity of 94–98%, which is comparable to CT and TEE; and superior to TTE or serologic biomarkers [109]. Black blood imaging can reveal the location and extent of the dissection flap and dynamic CE-CMRA can be used assess filling dynamics of the true and false lumen. 4D flow imaging can be used to visualize and quantify flow in both lumina as well as through any fenestrations (Fig. 4). Due to the outstanding soft tissue contrast, imaging of the aortic wall is a particular strength of CMR. Intramural hematoma (IMH) is a variant of dissection, where the false channel in the aortic wall is filled with thrombus. No primary dissection flap will be visible and two-lumen flow is absent. In IMH with hyperacute bleeding, T_2_-weighted images display hyperintense signal in the hematoma, whereas signal is isointense on T_1_-weighted images. After approximately first 24 h, the IMH will appear hyperintense on both T_1_- and T_2_-weighted images. These features help differentiate IMH from mural thrombus, which appears hypointense or isointense on both T_1_- and T_2_-weighted sequences [110]. The use of fat-saturation techniques is helpful to distinguish IMH from the mediastinal fat surrounding the aorta. Penetrating atherosclerotic ulcer is a form of dissection where there is intimal erosion with ulceration extending through the internal elastic lamina into the media and/or focal IMH [111, 112]. Penetrating atherosclerotic ulcer is a *cause* of aortic hematoma and should be distinguished from ulcer-like projection, which is a *consequence* of the hematoma or thrombus in the wall, and only appear after an initial IMH [113]. Differentiation of both entities is crucial since a penetrating atherosclerotic ulcer surrounded by an IMH has a higher risk of aortic rupture than an IMH complicated with a ulcer-like projection or localized dissection. A penetrating atherosclerotic ulcer with persistent pain, with an IMH or periaortic haemorrhage must be treated surgically or with thoracic endovascular aortic repair (TEVAR) [112]. CE-CMRA may show a focal defect in the arterial intima and, typically, deformation of the external aortic contour. T_1_- and T_2_-weighted sequences can demonstrate focal IMH as described above.

CMR is also increasingly used to identify aortic atheroma as a potential source of cerebral emboli in cryptogenic stroke. A combined protocol using T_1_-weighted bright blood, T_2_- and proton-density weighted black blood and 4D flow imaging was recently shown to be capable of depicting complex aortic plaque in the aortic arch and proximal descending aorta as well as potential embolization pathways from such plaques to the brain [114].

### Carotid and cervical arteries

Carotid artery CE-CMRA is a highly reliable technique to depict the presence and extent of atherosclerotic plaque formation in the aortic arch branch vessels and to measure the degree of carotid artery stenosis [115]. Advances in CMR hardware such as dedicated coils and the introduction of high spatial and temporal resolution CE-CMRA pulse sequences have enabled sub-millimeter isotropic voxel sizes in short imaging times using K-space view sharing techniques [116]. Venous overlap can be avoided by using centric K-space ordering. Promising results have also been obtained with non-contrast enhanced techniques [117].

### Pulmonary arteries

Advances in CMR hardware and pulse sequence design have enabled high-fidelity depiction of the pulmonary arteries and veins in short imaging times [118]. The most common acquired disease of the pulmonary arteries is pulmonary embolism (PE) and CMRA can function as an alternative to CTA to rule out PE. The prospective multicentre CE-CMRA for pulmonary embolism (PIOPED III) study found that a technically adequate protocol that combined pulmonary CMRA and CMR venography of the peripheral veins has a sensitivity of 92% and a specificity of 96% compared to the reference standard [119]. However, in this study a large proportion of examinations were technically inadequate due to motion artefacts and inadequate vascular opacification [120]. For this reason, CMRA of the pulmonary arteries should only be considered in at centers that routinely perform it and in patients in whom standard tests (e.g., CTA) are contraindicated. A more recent study that combined a non-contrast and CE protocol found CMR to be practically equivalent to pulmonary CTA in patients with suspected thromboembolism [121]. Pulmonary artery CMRA remains the technique of choice for integrated cardiopulmonary evaluation of acquired pulmonary artery stenosis [122], evaluation of pulmonary artery aneurysms [123] and dissection [124].

### Abdominal aorta, renal and mesenteric arteries

CE-CMRA remains the technique of choice for imaging the abdominal aorta and its branches. The ability to obtain high spatial resolution images of these vessels within a single breath hold enables accurate depiction of stenosis in the abdominal aorta, renal and mesenteric arteries [125]. CMRA is also well-suited to characterize aneurysmal disease of the abdominal aorta. The excellent soft tissue contrast facilitates detection and characterization of both the vascular lumen as well as mural thrombus. A combined CMRA and abdominal CMR protocol can even be used in patients with some types of abdominal aortic stents to assess the presence of endoleak after endovascular aortic aneurysm repair and has been shown to outperform multiphasic CTA for this purpose [104, 105].

For many years CE-CMRA has been an accepted modality to depict the renal arteries. Renal artery CMRA is used to visualize the number and course of the renal arteries prior to nephrectomy and dynamic CE sequences can be added to the imaging protocol to quantify renal perfusion [126]. However, since publication of the Cardiovascular Outcomes in Renal Atherosclerotic Lesions (CORAL) [127] and the Angioplasty and Stenting for Renal Artery Lesions (ASTRAL) [128] trials which both demonstrated that subjects with renal artery stenosis had similar outcomes whether randomized to optimal medical therapy alone or optimal medical therapy plus renal artery stenting, clinical interest in imaging renal artery stenosis has decreased. On the other hand, the introduction of renal sympathetic denervation [129, 130] has led to a renewed interest in imaging of renal artery anatomy and non-invasive imaging of renal function. The ability to simultaneously depict renal anatomy and physiology as well as cardiac function also allows unique insights into cardiorenal function [131]. Stenosis of the celiac trunk and mesenteric arteries can also be reliably diagnosed with CMRA techniques. Although CTA is preferred in the acute setting, both techniques can be used to depict the degree of atherosclerotic narrowing and presence and extent of collateral circulation, as well as other non-atherosclerotic vascular pathologies such as fibromuscular dysplasia and compression of the celiac trunk by a median arcuate ligament [132]. In the latter condition CMRA is preferred as it allows for obtaining both inspiratory and expiratory views of the celiac trunk without radiation burden.

### Lower extremity arteries

**Fig. 5:**
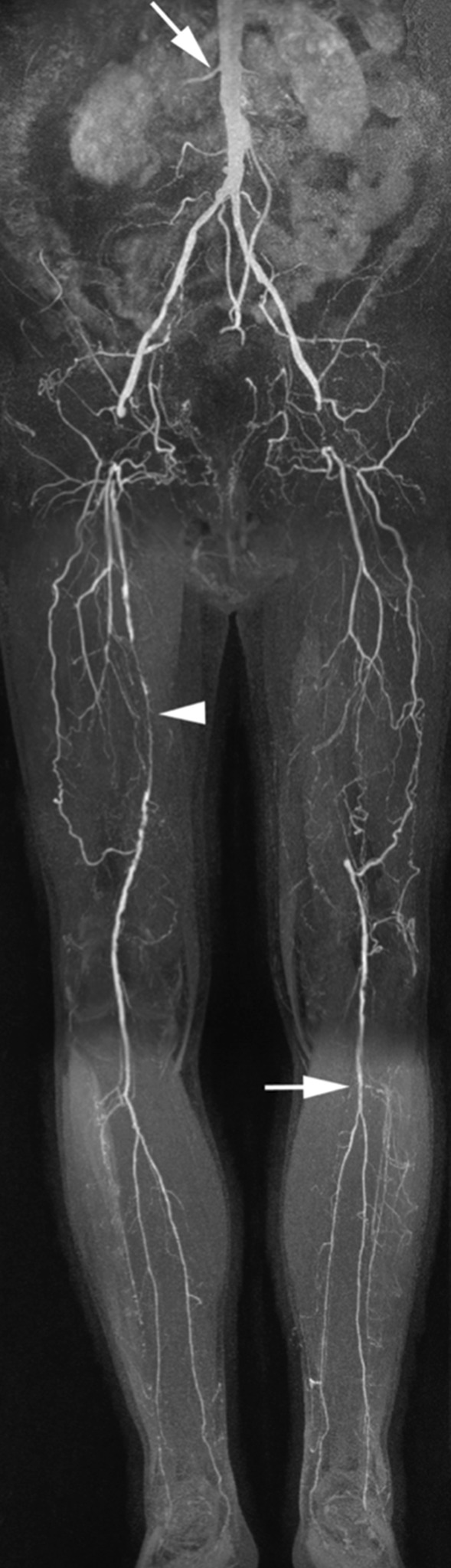
65-year-old man with bilateral Fontaine IIb peripheral arterial disease. There are bilateral common femoral artery occlusions bridged by collaterals, as well as an occluded superficial femoral artery in the left leg. Collateral arteries bridge the common femoral arteries. The right superficial femoral artery shows a high-grade stenosis (arrowhead). The tibioperoneal trunk and proximal posterior tibial artery are clearly patent (horizontal arrow). There is subtle narrowing of the proximal right renal artery (oblique arrow)

CMRA has been shown to be a highly reliable technique to depict the presence and extent of arterial narrowing in patients with intermittent claudication and chronic critical ischemia [133–136]. In most patients, atherosclerotic peripheral arterial occlusive disease is the underlying cause of arterial narrowing. The imaging protocol typically consists of acquisition of 3–4 FOVs or ‘steps’ during infusion of 0.1–0.2 mmol/kg contrast agent. Such protocols enable depiction of the peripheral vasculature from the aorta down to the feet with high vessel to background contrast [137–139]. The separate acquisitions are then ‘stitched’ together to provide a comprehensive overview of the peripheral vascular tree (Fig. 5). In patients with chronic critical ischemia the lower leg and pedal vasculature may be compromised by venous contamination. To avoid this problem, it is recommended to use a separate low-dose injection of contrast medium in combination with a time-resolved acquisition. Not only does this avoid the problem of venous overlay, but it also provides hemodynamic information about the flow direction in severely diseased arteries and it enables high-quality imaging of small, distal vessels in patients with differential flow in the lower extremities. To further optimize depiction of the small distal arteries it is desirable to use fat suppression. This can be done by subtraction of non-contrast-enhanced ‘mask’ images, or by using pulse sequences such as the modified Dixon technique [104]. The outstanding soft tissue contrast of CMR also enables diagnosis of alternative vascular pathologies that may lead to intermittent claudication such as popliteal entrapment, vasculitis, cystic adventitial disease, fibromuscular dysplasia and other more uncommon diseases [140].

Recently, significant advances have been made in non-contrast CMRA of the peripheral vessels. Promising results have been obtained with quiescent interval single shot (QISS) CMRA. In a study in 53 patients with suspected or known peripheral arterial disease the diagnostic performance of QISS CMRA was shown to be nearly equivalent to CE-CMRA and digital subtraction angiography [141]. The same investigators also demonstrated the ability to depict areas in the arterial vessel wall containing calcium deposits [142], which further enhances the attractiveness of CMR as an alternative to CT.

### Arterial wall imaging

The ability to null blood signal provides important opportunities for detection and characterization of vessel wall abnormalities with CMR. This does not only concern imaging of atherosclerotic plaque, but also inflammatory changes associated with vasculitis and rheumatological diseases. CMR vessel wall imaging has been shown to be capable of detecting, characterizing and quantifying atherosclerotic plaque in the aorta [143, 144], carotid arteries [145, 146], lower extremity arteries [147, 148] and even the coronary [149, 150] and intracranial arteries [151, 152]. The large FOV allows depiction of arterial wall changes over large anatomical trajectories. The imaging protocol typically consists T1-, T2- and proton density weighted images which enables identification of lipid-rich necrotic core and intra-plaque haemorrhage – the two most important features that portend plaque rupture and clinical sequelae –with high sensitivity and specificity [146]. More recently, combined angiographic and black blood imaging sequences have been described that allow fast, simultaneous imaging of the vasculature as well as the presence of intraplaque haemorrhage [153]. T1 and T2 vessel wall mapping protocols are now also becoming available and may remove some of the subjectivity of conventional T1- and T2-weighted sequences [154].

In patients with suspected or known large and medium vessel vasculitis, 3D fat-suppressed black-blood T1-weighted turbo spin-echo sequences have been of particular value to depict inflammatory changes such as post-contrast vessel wall enhancement in the aorta and its branches [144], extra- and intracranial carotid artery and its branches [155, 156], as well as the cerebral arteries [157].

### Pulse wave velocity

Early atherosclerotic changes in the arterial wall lead to outward remodelling and are not yet visible as luminal narrowing, which limits the utility of angiographic techniques. Pulse wave velocity (PWV) is a biomarker of arterial stiffness, which is known to increase long before the advent of stenosis. CMR is the technique of choice to measure PWV because it is a highly reliable for measurement of vessel length and vascular velocity waveforms at different locations in a vessel. The pulse wave can be understood as a wave superimposed on the flow/pressure waveform of the blood. This superimposed pulse wave accelerates and decelerates as it traverses distally in the vasculature relative to the stiffness of the vessel in a given segment [158]. PWV is arguably one of the earliest markers of atherosclerosis and alterations in aortic PWV have been shown to be related to the level of insulin resistance in children with type-1 diabetes mellitus [159], to blood pressure, body mass index and levels of expression of cellular adhesion molecules [160] in young adults [161], as well as cognitive decline in the elderly [162].

### Arterial reactivity

Another CMR based method to non-invasively assess vascular function is artery reactivity. Endothelial function can be examined non-invasively with stimuli which cause arterial vasodilation. Flow mediated dilation is used to examine endothelial function directly, by occluding usually the forearm using a blood pressure cuff inflated above systolic pressure for a standard time period. On release of the cuff, reactive hyperaemia causes increased endothelial shear and the release of nitric oxide which causes the brachial artery to dilate. Endothelial independent responses can also be tested by using glyceryl trinitrate, typically as a sublingual spray. Visualisation of brachial dilation with these stimuli was first described using ultrasound [163], but it may be difficult to ensure that the transducer is correctly positioned perpendicular to the artery and without movement, and that repeated measurements are made with good reproducibility. CMR techniques are considered to have advantages in both these areas and comparisons of CMR and ultrasound for accuracy and reproducibility favour CMR [164]. In addition to measuring brachial dilation, CMR can also measure flow changes directly in response to the standard stimuli [165, 166] and has been used to assess residual signs of vascular damage after repair of aortic coarctation [167]. Vascular reactivity can also be measured in lower extremity arteries and, more so than in the forearm arteries, has been shown to be progressively reduced with an increase in cardiovascular risk factors [168].

### Venous system

The main indications for imaging the central veins are assessment of suspected anatomical variants and follow-up of patients with CHD [169], mapping of pulmonary venous anatomy [170], vena cava superior syndrome [171] as well as assessment of the central veins prior to creation of upper extremity vascular access in patients with renal dysfunction [172]. CMR venography is also increasingly used to assess the peripheral venous system in patients with venous compression syndromes [173], venous anomalies [174], and suspected or known deep venous thrombosis of both the upper [175] and lower extremities [176].

Both CE and non-contrast CMRA techniques can be used for depiction of the venous system. For imaging of chest veins non contrast cardiac triggered and respiratory navigator-gated bSSFP techniques can provide excellent image quality in short imaging times [177] and is often sufficient to answer the clinical question. CE-CMR venography can be performed with both extracellular as well as blood pool contrast agents. The approximate intravascular half-life of extracellular chelates is around 60–120 s [178]. Thus, when a conventional extracellular contrast agent is used, rapid leakage of the contrast agent into the interstitial space will reduce enhancement of both the arteries and the veins shortly after injection. In order to obtain good quality images of the venous system it is therefore mandatory to initiate the acquisition immediately after the first pass of the contrast agent. An alternative strategy is to use the blood pool agent ferumoxytol, which has a much longer intravascular half-life, and thereby facilitates ultra-high spatial resolution depiction of both the arterial as well as the venous systems with voxel sizes about one order of magnitude smaller compared to conventional CMR vascular imaging techniques [179]. This contrast agent has been shown to be of high value in patients with CHD [180], imaging of central [181], abdominal [182] and peripheral veins [183].

## Coronary artery disease

Coronary artery disease (CAD) spans a broad range of acquired and congenital coronary artery abnormalities. The vast majority of events such as myocardial infarction (MI) result from atherosclerosis. CAD may be further classified as ‘macrovascular’—involving the epicardial segments of the coronary artery tree—or ‘microvascular’ where vasomotor, neurohormonal and other factors affect the coronary microcirculation. CMR has proven utility in addressing most aspects of CAD, illuminating mechanism of disease and guiding the selection of therapeutic strategies (Table 3).

**Table 3:** Indications for CMR in coronary artery disease

Indication	Class
1. Acute coronary syndromes	I
2. Chronic coronary artery disease	I
3. Myocardial infarction with non-obstructive coronary arteries (MINOCA)	I
4. Coronary artery anomalies	II

### Acute coronary syndromes

Patients with acute coronary syndrome (ACS) have myocardial ischemia or injury resulting from disruption to coronary blood flow. Assessment of a standard 12-lead electrocardiogram (ECG) at presentation yields 2 broad categories of ACS: ST-segment elevation myocardial infarction (STEMI) and non-ST elevation events (NSTEMI). Classically, STEMI results from complete thrombotic occlusion of a coronary artery segment, whereas NSTEMI may represent subtotal occlusive or erosive CAD. Recognition of STEMI mandates rapid, uniform deployment of community-to-catheterization laboratory evaluation and management aimed at recanalization of the occluded coronary artery. No modality including CMR has shown compelling utility to support slowing down established pathways by adding imaging between presentation and coronary intervention. After invasive coronary angiography, however, CMR with its workhorse imaging technique of late gadolinium enhancement (LGE) affords in vivo visualization of myocardial injury, and hypointense regions within infarct scar indicate microvascular obstruction (MO). CMR may inform post-STEMI care when complicated by heart failure, arrhythmias, or LV dysfunction. The transmural extent of myocardial damage by LGE is inversely related to the likelihood of functional recovery, with even worse prognosis conferred with the presence of MO [184]. T2-weighted imaging combined with LGE has been used to detail the extent of myocardium that has been salvaged after revascularization in patients with STEMI [185]. Fortunately, mechanical complications post-STEMI such as papillary muscle rupture, free wall rupture and post-infarct ventricular septal defect have diminished in the era of rapid catheter-based reperfusion. However, reperfusion myocardial injury and intramyocardial hemorrhage still occur and may contribute to residual risk [186]. The comprehensive nature of CMR can fully characterize the jeopardized region in aborted STEMI, while precisely assessing the complications such as RV infarction, post MI pericarditis, and thrombus formation [187–189]. Contemporary mapping of myocardial T1, T2 and T2* values along with traditional LGE imaging affords detailed characterization of cardiac muscle post-STEMI, affording insights into mechanisms of adverse remodeling and other downstream complications [190].

These techniques can be used to test novel approaches to preserve myocardium in STEMI, such as optimal timing of stent placement in patients undergoing aspiration thrombectomy [191]. Incorporating CMR’s precise myocardial biomarkers has accelerated the evaluation of a number of complementary therapeutic approaches. For instance, Bulluck and colleagues [192] evaluated treatment with a mineralocorticoid receptor antagonist drug intravenously at the time of primary percutaneous coronary intervention (PCI) and continued orally for 10 weeks after PCI; CMR was performed in the first week and again after 3 months. While mineralocorticoid receptor antagonist therapy did not reduce infarct size, LV remodeling did improve. Such CMR-enabled studies advance the evidence needed to improve post-STEMI outcomes.

NSTEMI patients are more heterogeneous compared to STEMI not only in ECG findings but also in time course of presentation and revascularization. Importantly, NSTEMI comprise the vast majority of all ACS and may represent a greater opportunity for CMR to guide management. For instance, T2 imaging prior to invasive angiography in patients with NSTEMI has been shown to delineate myocardium at risk of irreversible injury, predicting both presence of CAD requiring revascularization as well as greater risk of adverse outcomes [193]. Recent trials have confirmed no advantage of early (within a few hours) vs. usual (within 72 h) timing of invasive coronary angiography (ICA) in the aggregate NSTEMI population lacking high risk features [194]. These data encourage feasibility of further trials that incorporate CMR into the evaluation of patients with NSTEMI, particularly as novel strategies emerge to protect myocardium from both ischemic as well as reperfusion injury. Incorporating CMR after angiography and randomization to either thrombectomy or standard PCI in NSTEMI, Thiele and colleagues [195] found that combining aspiration thrombectomy with PCI in NSTEMI with a thrombus-containing lesion did not reduce MO.

### Chronic coronary artery disease

**Fig. 6:**
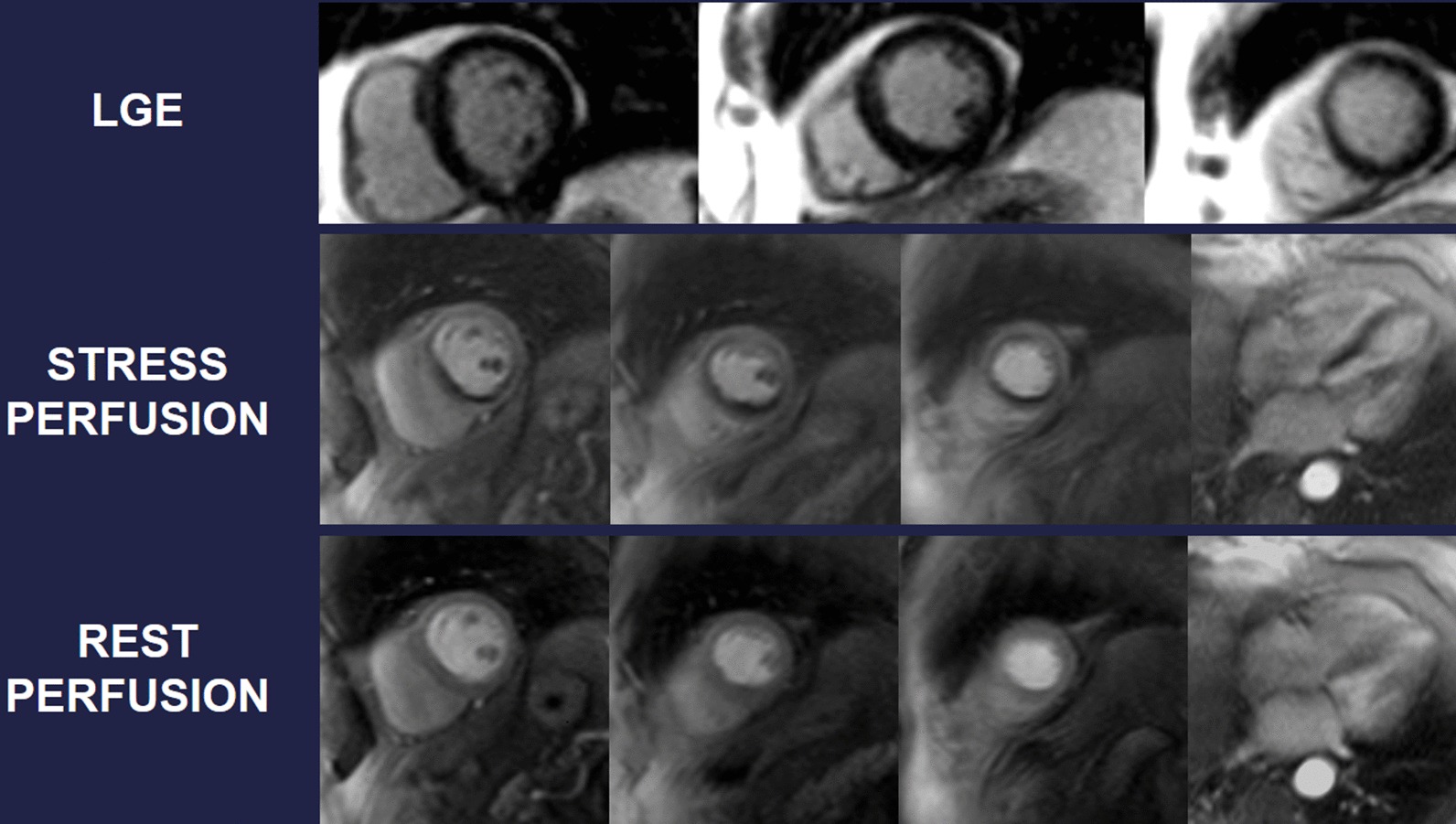
A 75 year-old male with hypertension and hyperlipidemia reported exertional dyspnea during rehabilitation post-stroke. Transthoracic echocardiography (TTE) showed mild left ventricular (LV) systolic dysfunction, and he had difficulty walking. Given concern for myocardial ischemia, he was referred for CMR with stress. Late gadolinium enhancement (LGE) demonstrates a small subendocardial infarct in the basal inferior wall, while myocardial perfusion acquired during adenosine infusion (stress) shows extensive perfusion abnormality that mostly resolves on resting perfusion imaging. Cine CMR demonstrates mild segmental LV dysfunction. These findings prompted invasive coronary angiography that showed high-grade multivessel coronary artery disease (CAD); post-revascularization, functional capacity improved

Symptomatic patients with known or suspected stable CAD may require evidence that symptoms are a result of CAD. Delineation of ischemic myocardium with stress myocardial perfusion imaging can guide coronary artery revascularization, targeting ischemic segments towards relief of symptoms (Fig. 6). Several head-to-head trials now endorse stress CMR with vasodilator perfusion as a more accurate modality in evaluating the symptomatic CAD patient [196, 197], with better utilization of costly resources like ICA [198]. A subsequent substudy of CE-MARC indicated greater accuracy in left main stem or equivalent CAD, recognizing grave consequences in missing such disease [199] and the MR-INFORM trial puts stress CMR on equal footing as assessment of what many deem the gold standard for CAD severity – invasive fractional flow reserve [200].

In an era of more complex questions regarding native coronary artery or bypass graft anatomy amidst scarred vs viable myocardium, CMR has distinct advantages over other modalities. While single photon emission tomography (SPECT) may infer the presence of infarct scar in patients with ‘fixed defects’ e.g. reduced tracer uptake on both rest and stress perfusion images, having both LGE and perfusion imaging techniques allows CMR to better distinguish scar from hibernating myocardium [201].

For individuals presenting in the acute setting with symptoms of intermediate CAD likelihood, stress perfusion CMR accurately identifies disease and safely facilitates discharge in patients with normal results, at lower cost when compared to standard inpatient evaluation with ICA [202].

The International Study of Comparative Health Effectiveness with Medical and Invasive Approaches, or ISCHEMIA trial, asked—how does routine invasive therapy improve outcomes compare to optimal medical therapy in patients with stable CAD and moderate to severe myocardial ischemia on noninvasive stress testing? While analysis from the small subgroup of subjects whose testing included stress CMR is underway, the overall trial results from over 5,000 patients randomized across 320 centers in 37 countries followed for a little over 3 years are relevant to today’s CMR practice. With a cohort of over three-fourths male patients with a significant burden of diabetes (41.8%), prior MI (19.2%) and preserved LV ejection fraction (LVEF) (median 60%), the primary outcome of cardiovascular death, MI, resuscitated cardiac arrest, or hospitalization for unstable angina or heart failure was similar among routine invasive (13.3%) and medical therapy (15.5%) groups (p = 0.34). These results endorse use of an accurate, cost-effective and noninvasive modality such as stress CMR in such patients in place of invasive evaluation.

### Myocardial infarction with non-obstructive coronary arteries (MINOCA)

Myocardium may suffer infarction from causes other than obstructive CAD such as plaque erosion, spontaneous coronary artery dissection, coronary artery vasospasm, and coronary artery embolization. CMR is typically called upon after ICA has not provided a definitive anatomic abnormality, and when distinction of MI from myocarditis may be difficult by symptoms such as chest pain with biomarker evidence of myocardial injury. Occasionally, the angiogram is reviewed after CMR shows the classic signature of MI—subendocardial pattern of damage by LGE—and subtle invasive coronary angiography findings of erosive plaque or dissection are found retrospectively. In this setting, LGE unequivocally identifies the downstream myocardial damage as ischemic vs. inflammatory (i.e. subendocardial vs. epicardial injury) [203] making CMR central per recent guidelines from a broad international writing group in the very definition of MI [204].

Dastidar and colleagues added significant evidence supporting CMR’s utility in both diagnosis and prognostic assessment in patients with myocardial infarction with non-obstructive coronary arteries (MINOCA) [205]. CMR—including cine, T2, and LGE imaging—was performed in 388 patients approximately 1 month after emergent or urgent invasive coronary angiography and showed nonobstructive CAD in the face of evident myocardial damage by elevation in blood troponin-T levels. The diagnosis of MINOCA was further refined by CMR as myocarditis in 25%, myocardial infarction in 25%, and cardiomyopathy in 25%. With 5.7% of patients suffering mortality after an average of 3.5 years, CMR diagnosis of cardiomyopathy combined with ECG presence of ST elevation were the only significant predictors of mortality in multivariable regression analysis. Importantly, such a diagnosis would be missed by modalities without CMR’s ability to identify myocardial disease, and consideration of appropriate treatment to improve outcomes in cardiomyopathy could be neglected. Such findings underscore the essential role of CMR in MINOCA.

### Coronary anomalies

A contemporary understanding of coronary artery development sheds light on the range of anomalies that may result including anomalous connection, intrinsic abnormality such as non-atherosclerotic ectasia or ostial atresia, and anomalous myocardial/coronary artery interaction [206]. While infrequently detected in adults undergoing invasive coronary angiography, coronary anomalies contribute to sudden death in young individuals, especially athletes, and may also occur in conjunction with other congenital abnormalities. The volumetric nature of CMR makes it ideal in visualizing the proximal course that may not be apparent by two-dimensional projection X-ray angiography. CMRA easily identifies inter-arterial (traveling between the ascending aorta and pulmonary artery) vs. retroaortic coronary anomalies, with greater concern regarding sudden death risk in the former [57]. While other modalities such as coronary CTA offer more reliable imaging of the mid and distal segments of the coronary artery tree, such distinction of the proximal anatomy is well within reach of CMR without requiring ionizing radiation. Navigator-triggered noncontrast coronary CMRA further removes the requirement for exogenous iodinated contrast to image the proximal course of the coronary arteries [207].

Ongoing and future clinical trials will hopefully illuminate novel CAD treatment approaches informed by CMR, particularly with mechanistically relevant endpoints to test efficacy.

### Kawasaki Disease

Kawasaki Disease is the prototypical systemic vasculitis that affects the coronary arteries. A disease with pediatric predilection, Kawasaki Disease typically presents with persistent fever and rash. Its potential for inflammation and aneurysm formation of the coronary arteries and ensuing myocardial damage typically mandates cardiovascular assessment, both acutely as well as in convalescence. CMR is ideal for comprehensive coronary and myocardial assessment in patients with Kawasaki Disease. Mavrogeni and colleagues offer a cogent summary of recommended CMR-based assessment during acute and chronic phases of the disease well-suited for techniques like coronary CMRA, functional assessment, perfusion with or without stress, and LGE imaging [208]. A recent quantitative CMR study, including circumferential strain measurements, showed changes like fibrotic myocardial remodeling beyond infarcted tissue in 19, mostly male, children with Kawasaki Disease [209].

These findings in a relatively rare disease are urgently relevant as the world struggles to respond to a Kawasaki Disease-like disease in children linked to the COVID-19 pandemic caused by severe acute respiratory syndrome coronary virus 2 (SARS-CoV-2) [210]. Systematic CMR studies are needed in children and convalescent adults to understand the long-term sequelae related to this increasingly recognized complication of COVID-19.

## Myocarditis and other cardiomyopathies

The cardiomyopathies are commonly understood as primary myocardial diseases, that are not caused by CAD, hypertension, valvular or CHD [211]. Importantly, cardiomyopathies are often characterized by tissue abnormalities that reflect chronic injury, infiltration, or abnormal storage of molecules [211]. These pathologies lead to signal intensity changes in CMR images before or after contrast agent injection. Their signal behaviours and regional distribution patterns often represent important complementary diagnostic information [212]. Yet, often the findings obtained by CMR alone is sufficient for establishing a diagnosis. Over the recent years, CMR has been increasingly accepted as a critically important tool in cardiovascular disease management [213] (Table 4).

**Table 4:** Indications for CMR of cardiomyopathies

Indication	Class
1. Dilated cardiomyopathy	I
2. Myocarditis	I
3. Hypertrophic cardiomyopathy	I
4. Arrhythmogenic cardiomyopathy	I
5. Cardiac amyloidosis	I
6. Myocardial iron overload	I
7. Left-ventricular noncompaction	I
8. Fabry’s disease	I
9. Cardiac sarcoidosis	I
10. Stress-induced (Takotsubo) cardiomyopathy	I
11. Endomyocardial fibrosis	I
12. Restrictive cardiomyopathy	II
13. Chemotherapy induced CMP	II
14. Athlete’s heart	II

CMR is widely accepted as the non-invasive gold standard for quantifying biventricular volumes, myocardial mass as well as regional/global systolic function [214]. LV function can be assessed using simplified CMR approaches such as biplanar long axis views [215] or rotational long axis views [216], yet typically short axis stacks of both ventricles are applied [217, 218]. There is consensus about the methods of the quantitative assessment of the heart in CMR images [219]. Normal values have been published for the LV [218, 220] and RV [221].

CMR is the most appropriate non-invasive method to assess tissue characteristics in vivo. The fundamental contrast-generating principle of CMR is rooted in the close relationship between the magnetic properties of tissue and its molecular composition. These predict the results of myocardial mapping and affect the signal intensity in standard CMR images. Standard CMR techniques include contrast-enhanced T1-weighted CMR after iv administration of contrast agents, typically GBCAs. These techniques have been successfully applied to visualize necrosis, scar, infiltration, inflammation, or intraventricular thrombi, while edema-sensitive T2-weighted CMR images can help identify acute injury [222]. T2*-weighted imaging have proven useful in the detection of myocardial hemorrhage [223] and thrombi [224]. Myocardial mapping allows for directly measuring the change of magnetic properties as expressed by the magnetic relaxation times native T1, T2, T2*, and the extracellular volume (ECV) derived from post contrast T1 [225].

### Dilated cardiomyopathy

Dilated cardiomyopathy (DCM) is characterized by ventricular dilatation, global systolic dysfunction and often accompanied by global myocardial fibrosis. CMR can accurately quantify volumes as well as regional and global LV function including wall thickening, and wall stress [226]. RV volumes, morphology, and function can usually be better assessed by CMR than with TTE [227]. In patients with DCM, LGE imaging can visualize regional fibrosis that allows for discriminating non-ischemic DCM from ischemic CMP by the invariably predominant subendocardial involvement [228]. An intramural layer of bright signal intensity, typically involving the basal anteroseptal segment, also known as “midwall stripe”, is found in about one quarter of patients with DCM patients and is a predictor of sudden death and ventricular arrhythmia [229]. This sign, albeit not specific for non-ischemic cardiomyopathy (CMP) may therefore be useful in the decision-making related to the implantation of implantable cardioverter-defibrillators. LGE imaging is also useful in the follow-up of patients with DCM. Myocardial mapping appears useful in patients with DCM [230], possible as an early marker [231].

### Myocarditis

CMR is regarded as the most useful non-invasive tool to assess for myocarditis [232]. It adds diagnostic value to a standard clinical follow-up [233] and its use increases the observed incidence of myocarditis [234]. However, it remains important to keep in mind that myocardial inflammation is not specific to viral myocarditis but also occurs in other acute myocardial diseases such as acute MI, acute cardiotoxicity, immune checkpoint inhibitor myocarditis or stress-induced Takotsubo cardiomyopathy. A recent expert consensus document provides guidance on the CMR techniques and their role in non-ischemic myocardial inflammation [235]. Although ventricular volumes, morphology and function often remain normal and thus their quantitative assessment is less important than in other non-ischemic cardiomyopathies, CMR may be useful by accurately quantifying these parameters at acute presentation and during follow-up.

**Fig. 7:**
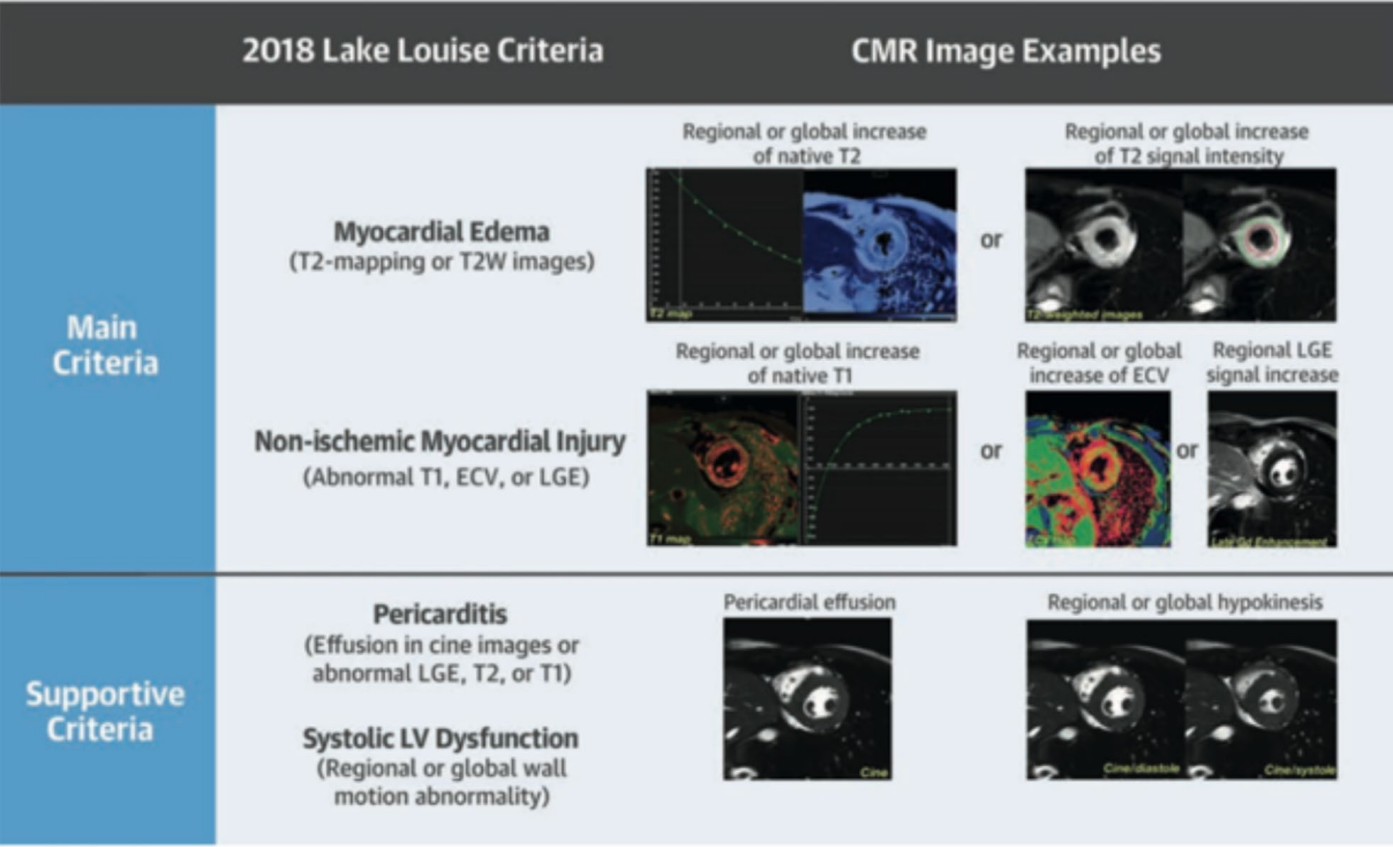
CMR criteria for non-ischemic myocardial inflammation (“Lake Louise Criteria”)

The diagnostic targets of CMR include edema and an increased extracellular space caused by necrosis or scar. The recently updated *Lake Louise Criteria for CMR in Non-Ischemic Myocardial Inflammation* recommend to assess them using T1-based and T2-based markers that detect myocardial edema and myocardial injury [235]. Figure 7 shows an overview of the criteria.

Increased signal intensity in T2-weighted imaging reflects inflammation-related myocardial edema [236, 237]. Patients with myocarditis typically show a regional or global signal intensity increase [237]. Edema may also be found in patients with DCM [238]. Irreversible myocardial injury can visualized in LGE images as areas with increased signal intensity with a non-ischemic regional distribution pattern [237, 239], with good agreement to histopathology [240]. LGE however has a limited sensitivity to detect myocarditis and is not specific to the acuity of the disease [235, 241]. The updated diagnostic criteria in suspected non-ischemic myocardial inflammation include (a) evidence for myocardial edema as shown by either an increased signal intensity in T2-weighted CMR images or an increased T2, and (b) evidence for myocardial injury as shown by either an increased myocardial T1, an increased myocardial ECV, or LGE in a non-ischemic regional distribution pattern [235]. Myocarditis is typically benign and myocardial edema disappears within weeks [241], whereas irreversible injury results in scars with persisting LGE.

### Hypertrophic cardiomyopathy

The hallmarks of hypertrophic cardiomyopathy (HCM) include an inadequate, mostly asymmetric increase of wall thickness and typically increased LV mass, associated with structural abnormalities, regional fibrosis, and LV outflow tract (LVOT) obstruction. All these markers can be quantitatively assessed by CMR. Myocardial wall thickness can be measured with more confidence than with TTE [242]. Of note, global LV mass may be normal, even in cases with marked regional hypertrophy [243]. Myocardial crypts have been associated with HCM [244], even in the absence of LV hypertrophy [245]. An overlap with the phenotype of LV non-compaction (LVNC) is being discussed. Abnormal global longitudinal strain is a marker for an impaired prognosis [246].

In HCM, the diagnostic workup can be significantly improved by CMR tissue characterization. Areas of marked hypertrophy often show regional fibrosis as focal areas with high signal intensity in LGE images [247, 248]. Furthermore, they occur at the insertion areas of the RV, likely due to increased mechanical stress in combination with relative coronary insufficiency in hypertrophied regions. LGE in HCM is associated with the risk for heart failure [249] and sudden cardiac death [250]. More definitive data are expected soon from the 3500 patient Hypertrophic Cardiomyopathy Registry (HCMR) trial [251]. Edema associated with HCM can be visualized by T2-weighted sequences, often co-localized with LV hypertrophy and irreversible injury [252].

Another diagnostic target in the clinical phenotyping of HCM is the presence and hemodynamic relevance of LVOT obstruction. This can be assessed by planimetric evaluation of the LVOT area [253], a parameter that appears to be more robust than the pressure gradient derived from 2D or 4D flow imaging [254]. In-plane flow imaging can be helpful to localise the peak LVOT velocity, but velocity quantitation can be difficult as the accuracy of flow mapping is often reduced in higher degrees of LVOT obstruction, due to the very narrow, turbulent jets with high acceleration.

CMR is suitable for monitoring functional and morphological changes after therapeutic interventions [253]. More recently, myocardial relaxation time mapping has also been applied to HCM [255] and helps improving our understanding of the interaction between morphology and function with tissue characteristics [256]. It is clinically important to distinguish pathological LV hypertrophy from athlete’s heart. This can be achieved by CMR using functional indices [257] or, as recently shown by the verification of a normal or even decreased proportional amount of extracellular space. Pathological hypertrophy is associated with an increased ECV, while athlete’s heart is not [258].

### Arrhythmogenic cardiomyopathy

Arrhythmogenic cardiomyopathy is a cause of sudden death, especially in young people. As it is associated with morphological and functional abnormalities of the RV, CMR is a preferred tool for adding diagnostic information to other known criteria derived from family history, ECG, and biopsies [259–261]. While the assessment of RV free wall fat is not used anymore, morphological and functional abnormalities serve as markers [262]. The original ESC Task Force criteria from 1994 [263] have been modified in 2010 to increase sensitivity [264]. Subsequent studies however have shown that sensitivity may be even lower, possibly because microaneurysms in the absence of RV dilatation have been removed as a marker [265]. Other abnormalities observable by CMR include trabecular hypertrophy and a irregular dilatation with trabecular hypertrophy of the basal RV wall (“accordion sign”) [262].

The assessment of the RV is often complicated by large interindividual differences of the anatomy, its irregular shape and difficulties in visualizing the connected valves by standard views. As arrhythmogenic cardiomyopathy is also associated with fibro-fatty or fatty degeneration of the myocardium, many studies have attempted to establish new diagnostic criteria based on tissue alone. While areas with bright signal intensity have been described in the RV or LV free walls, RV wall tissue characterization has been notoriously difficult because of the thinness of the RV wall and the resulting susceptibility to partial volume effects, that render tissue characterization techniques unreliable.

### Cardiac amyloidosis

In patients with amyloidosis, cardiac involvement typically predicts their outcome. The main purpose of CMR therefore is the exclusion or confirmation of cardiac amyloid. While cine images are important to assess for the typical combination of concentric LV hypertrophy with reduced compliance and restriction, often combined with atrial dilatation and thickening of the valves and the atrial wall, the most important contribution of CMR is based on its ability to demonstrate tissue characteristics. The deposition of amyloid protein in the myocardium results in a marked increase of native myocardial T1 [266] and to a rapid and strong uptake of gadolinium with a typical rapid clearance of the blood from the contrast agent [267]. Because the increase of native T1 is extensive, amyloid can in many patients be demonstrated by myocardial mapping, without the use of a GBCA [225]. Of the mapping variables, the ECV has been shown to most closely mirror the amyloid burden and the response to treatment, probably because T1 and T2 effects vary with variable presence of inflammation in this condition.

### Myocardial iron overload

Suspected myocardial iron deposition in patients undergoing repeated transfusions has become one of the most important applications of CMR, especially in geographical areas with a high incidence of thalassaemia, although this disease is now present worldwide due to immigration. As heart failure is the most frequent cause of death in patients with untreated iron overload, CMR can play a critical role in managing such patients. Because iron acts as a paramagnetic agent, its presence can be detected by its marked shortening effect on myocardial T2*. Since the introduction of this technique [268], the approach has been shown to be clinically very useful [269] and has greatly reduced cardiac mortality by ensuring early heart-tailored iron chelation treatment is started in patients who have been shown to be at high risk (those with T2* < 10 ms) [270]. CMR is directed toward the detection of myocardial iron deposits [271]. The T2* CMR technique has been validated in randomised controlled trials and is calibrated against human myocardium [272]. Recently, native T1 has been proposed as a marker of cardiac iron [273], but remains controversial because of lack of validation against outcomes and human tissue [274].

### Left-ventricular noncompaction

Left-ventricular noncompaction (LVNC) cardiomyopathy is characterized by LV dilatation, hypokinesis, and an abnormally high proportion of trabecular, non-compacted myocardium in combination with a thin compact wall. Initially described by TTE [275], the disease is associated with sudden cardiac death, yet is insufficiently understood [276, 277]. A genetic and phenotypic overlap with HCM is likely [278]. Generally, a wall thickness ratio of noncompacted over compacted wall of 2.3 or higher was proposed to verify LVNC [279], yet the specificity of non-compacted LV myocardium for this disease has been questioned [280, 281]. More recently, a cutoff of 3 was proposed [282]. Other studies proposed a cutoff of 20% of trabecular proportion of the entire LV myocardial mass [283] or a 35% proportion of non-compacted volume of 35% of the LV volume [284]. LGE was not found to be a strong diagnostic marker [285] although a recent meta-analysis found that patients with LVNC but without LGE have a better prognosis than those with LGE. When LGE is negative and global systolic function is preserved, no hard cardiac events are to be expected [286]. While a clearly abnormal amount of trabeculation in combination with a thin, hypocontractile wall allows for establishing the diagnosis, care should be taken not to overcall LVNC [287].

### Anderson-Fabry disease

**Fig. 8:**
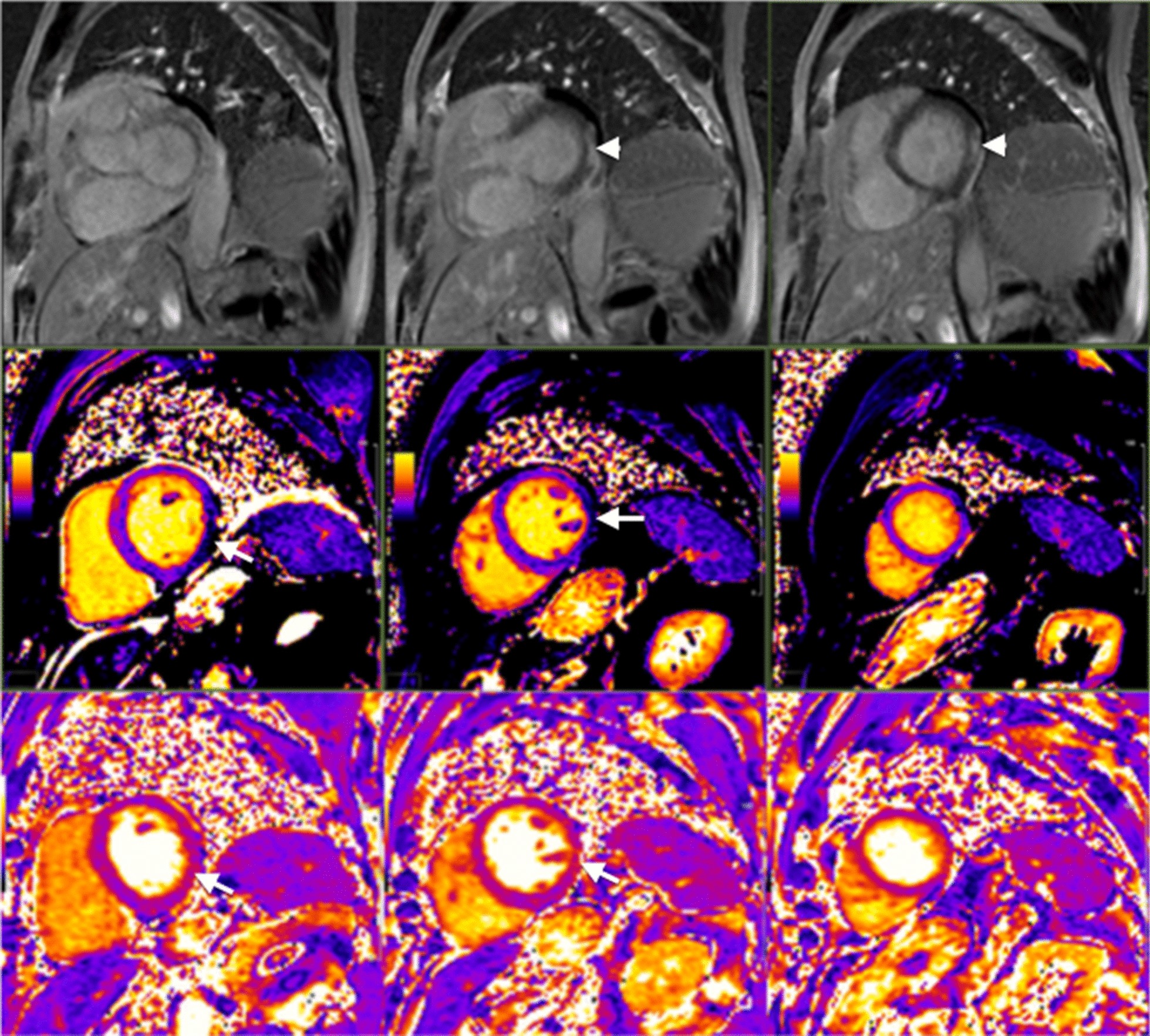
CMR in a patient with Anderson-Fabry Disease and associated myocardial inflammation. Upper row: LGE images showing inferolateral subepicardial LGE (arrowheads). Middle row: Native T1 maps showing a global decrease of native T1, specifically in the inferolateral wall (arrows). Lower row: T2 maps with increased myocardial T2, including the inferolateral wall, co-located with the areas that showed a low T1 (arrows)

Anderson-Fabry disease is caused by an enzyme defect leading to the accumulation of sphingolipids. Because the morphological appearance in myocardial involvement is indistinguishable from other forms of symmetric hypertrophy, the ability of CMR to identify sphingolipid storage in the myocardium has become a key diagnostic tool [288, 289]. Lipids lead to a decrease of native T1 and thus, concentric LV hypertrophy with a globally reduced T1 allows for the diagnosis of Anderson-Fabry Disease [225, 290]. LGE images can demonstrate layers of LGE, typically in the lateral wall [291]. Recently, evidence was provided that Anderson-Fabry Disease can present with edematous inflammation that can be demonstrated by increased T2 values [292]. Figure 8 shows a case of Anderson-Fabry disease with basal lateral LGE, a decreased T1 and an increased T2.

### Cardiac sarcoidosis

**Fig. 9:**
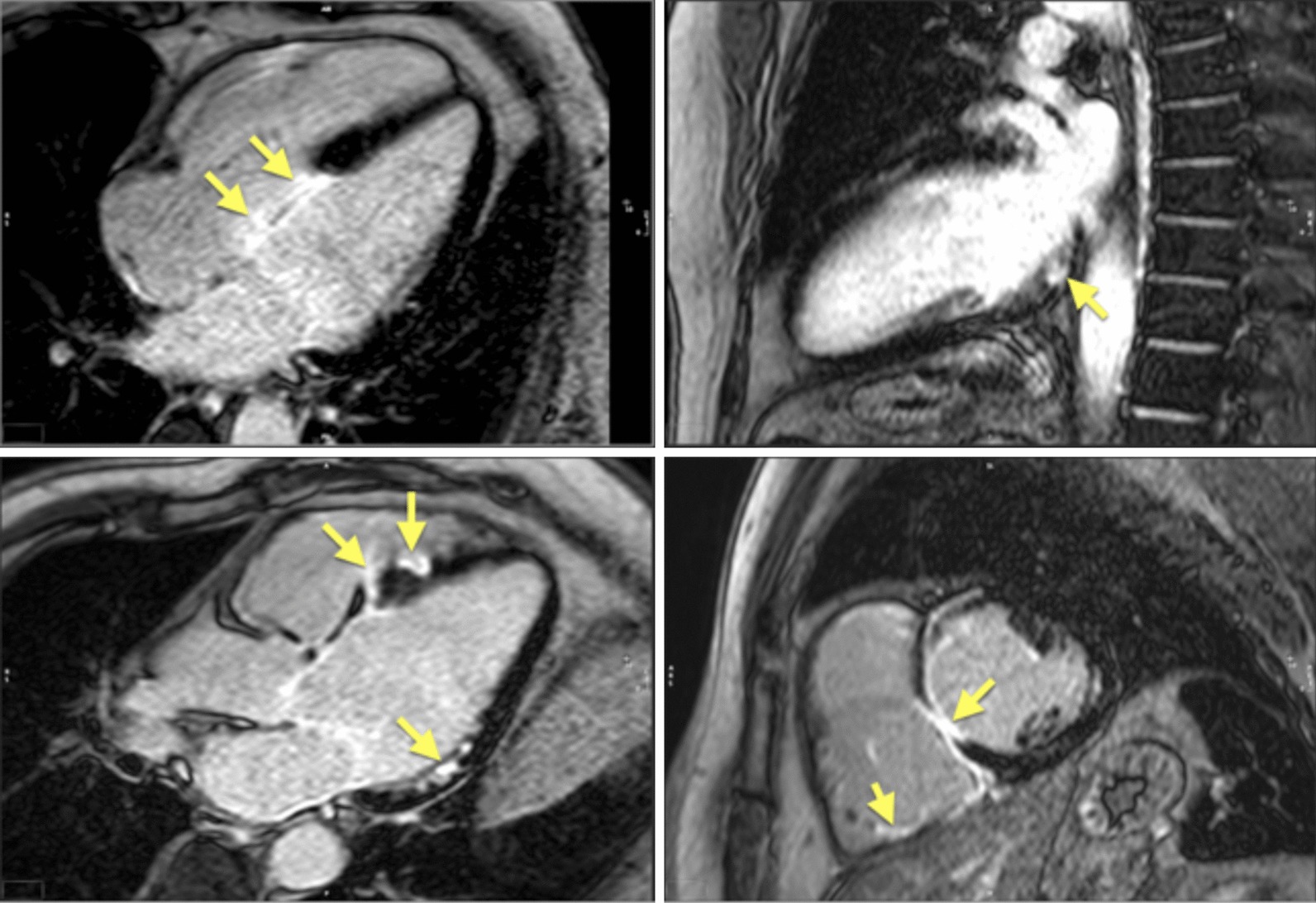
LGE images in a patient with pulmonary sarcoidosis and extensive cardiac involvement. There are several focal non-subendocardial based LGE areas with infiltration (arrows) in a variable pattern of transmural distribution

In patients with pulmonary or extracardiac sarcoidosis, CMR should be used to verify or exclude myocardial infiltration and associated inflammation [293–295] as cardiac involvement is a frequent cause of death in this population [296]. Next to LV dilatation and often global systolic dysfunction, the patterns of the regional distribution of myocardial lesions varies substantially, even in the same individual and may be intramural, transmural, subendocardial or subepicardial (Fig. 9) [254, 297, 298]. Edema is often present and identifiable on T2-weighted images [254, 299].

### Stress-induced (Takotsubo) cardiomyopathy

Stress-induced cardiomyopathy, also known as Takotsubo cardiomyopathy, is characterized by a reversible, extensive systolic wall motion abnormality, typically primarily involving the mid and apical LV [300, 301]. As with other imaging techniques, CMR can visualize systolic “ballooning” of the LV. On a tissue level, the hallmark of stress-induced cardiomyopathy however is transmural extensive edema [302]. LGE is rarely observed. Results of an international multi-center trial indicate that the CMR findings are consistent with acute inflammation (likely induced by catecholamines) and that the combination of the typical wall motion abnormality with extensive edema in the absence of LGE may allow for establishing the diagnosis [303, 304].

### Endomyocardial fibrosis

Endomyocardial fibrosis leads to a mainly apical concentric wall thickening, caused by extensive subendocardial fibrosis, frequently associated with an apical intraventricular thrombus. CMR is used to assess for ventricular volumes (typically small), and systolic dysfunction. As a more specific finding, endomyocardial fibrosis can be verified by LGE images [305, 306]. This pattern has also been described in patients with Churg-Strauss Syndrome [307].

### Restrictive cardiomyopathy

In patients with suspected myocardial restriction, CMR assessment helps in verifying the diagnosis by demonstrating small ventricles in combination with enlarged atria [308–311]. Furthermore, CMR allows for exclusion of constrictive pericarditis by demonstration absence of pericardial thickening [312] or by the absence of septal flattening during deep inspiration (real-time cine CMR sequences) [313]. Data however are scarce as this is a relatively rare entity.

## Pericardial disease

The pericardium—a virtual, sub-atmospheric space under normal circumstances—can be the location and cause of significant cardiac morbidity and mortality. Standard cardiac imaging modalities such as TTE, not infrequently fail to adequately investigate this part of the heart. Other imaging techniques such as CT provide an excellent morphologic view on the pericardium but are limited to depict the hemodynamic impact on pericardial disease on the heart. CMR has evolved to one of the preferred modalities for pericardial imaging [314, 315]. Using a series of CMR sequences, a combined morphologic-functional view on the heart and pericardium is achieved, providing adequate information with regard to the condition of pericardium, hemodynamic consequences on the heart and the presence of concomitant or superimposed myocardial/valvular disease [315] (Table 5). Morphologic sequences (e.g. spin-echo based sequences) allow detailed description of pericardial anatomy and its relation with the heart and surrounding anatomic structures. Pericardial tissue characterization—pericardial edema/inflammation/fibrosis—is achieved using a combination of non-contrast and CE sequences. It should be mentioned that for identification of pericardial calcium, CT is the best imaging modality [316]. bSSFP cine CMR allows to evaluate hemodynamic consequences, i.e. cardiac tamponade and constriction. In particular real-time bSSFP cine CMR during free breathing is able to depict pathologic ventricular coupling in patients with constrictive pericarditis [317]. Finally, phase-encoded cine CMR is of interest to study the cardiac inflow patterns, and thus to obtain information with regard to diastolic function [318].

**Table 5:** Indications for CMR in pericardial disease

Indication	Class
1. Pericardial effusions	III
2. Pericardial inflammation	I
3. Pericardial constriction	I
4. Congenital anomalies of the pericardium	I

### Pericardial effusions

Together with CT, CMR is likely the preferred modality to diagnose and to differentiate pericardial effusion. As CMR is not limited as echocardiography by the need of an adequate acoustic window, the entire pericardium can be satisfactorily investigated allowing to depict small or loculated effusions or to describe complex configurations [314, 315]. Typically, pericardial effusion yields high signal intensity at bSSFP cine CMR, allowing to depict for example fibrinous strands [315]. Although TTE is the first-line imaging modality in patients with cardiac tamponade, right atrial/ventricular collapse can be shown at bSSFP cine CMR as well, for example in patients with chronic pericardial effusions. As pericardial effusion frequently occurs in the setting of inflammatory pericarditis, and rarely in constrictive pericarditis (‘effusive–constrictive’ forms), CMR is highly useful to differentiate simple effusions from pericarditis-related effusions.

### Pericardial inflammation

Inflammatory pericarditis can be isolated or part of systemic disease. TTE diagnosis relies on the depiction of pericardial effusion. However, many patients have no (the so-called “dry pericarditis”) or only physiologic amounts of pericardial effusion [319]. As pericarditis is histologically characterized by thickening, edema, increased vascularity, and inflammation of the pericardial layers, the alterations can be used to depict pericardial inflammation at CMR [320]. Edema of the pericardial layers yields high-signal at T2-weighted spin-echo sequences. Also, at LGE CMR imaging, pericardial inflammation is characterized by strong pericardial enhancement [321]. In cases of effusive-inflammatory pericarditis, LGE CMR imaging allows to differentiate the inflammatory component (‘bright’) from the effusive component (‘dark’). CMR allows as well to depict associated inflammatory myocarditis. In patients with recent MI, CMR is well suited to depict epistenocardiac pericarditis [322].

### Pericardial constriction

**Fig. 10:**
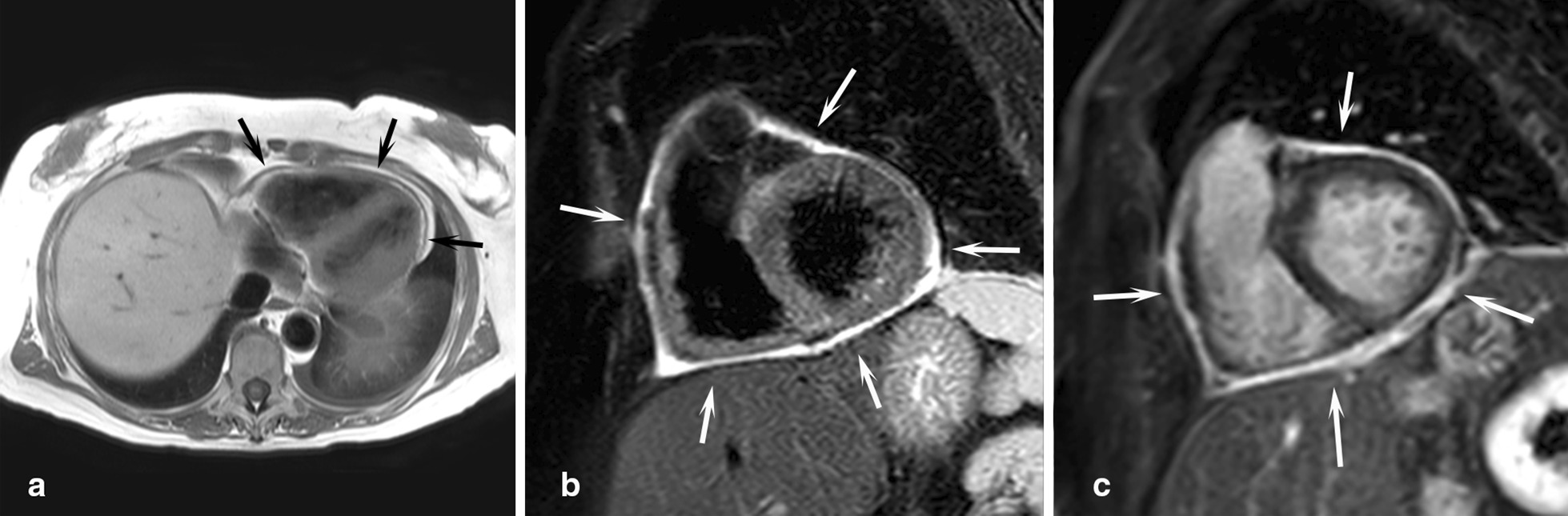
62-year-old woman with clinical presentation of Dressler’s syndrome following mitral and tricuspid valve replacement. Axial T1-weighted FSE imaging shows mildly thickening pericardium (**a**, black arrows). Short-axis T2-weighted fast spin echo imaging shows diffuse hyperintense appearance of the pericardium (**b**, white arrows). Late gadolinium enhancement imaging in cardiac short-axis shows diffuse strong pericardial enhancement (**c**, white arrows). CMR findings are highly suggestive of inflammatory pericarditis without evidence of associated pericardial effusion (“dry pericarditis”)

**Fig. 11:**
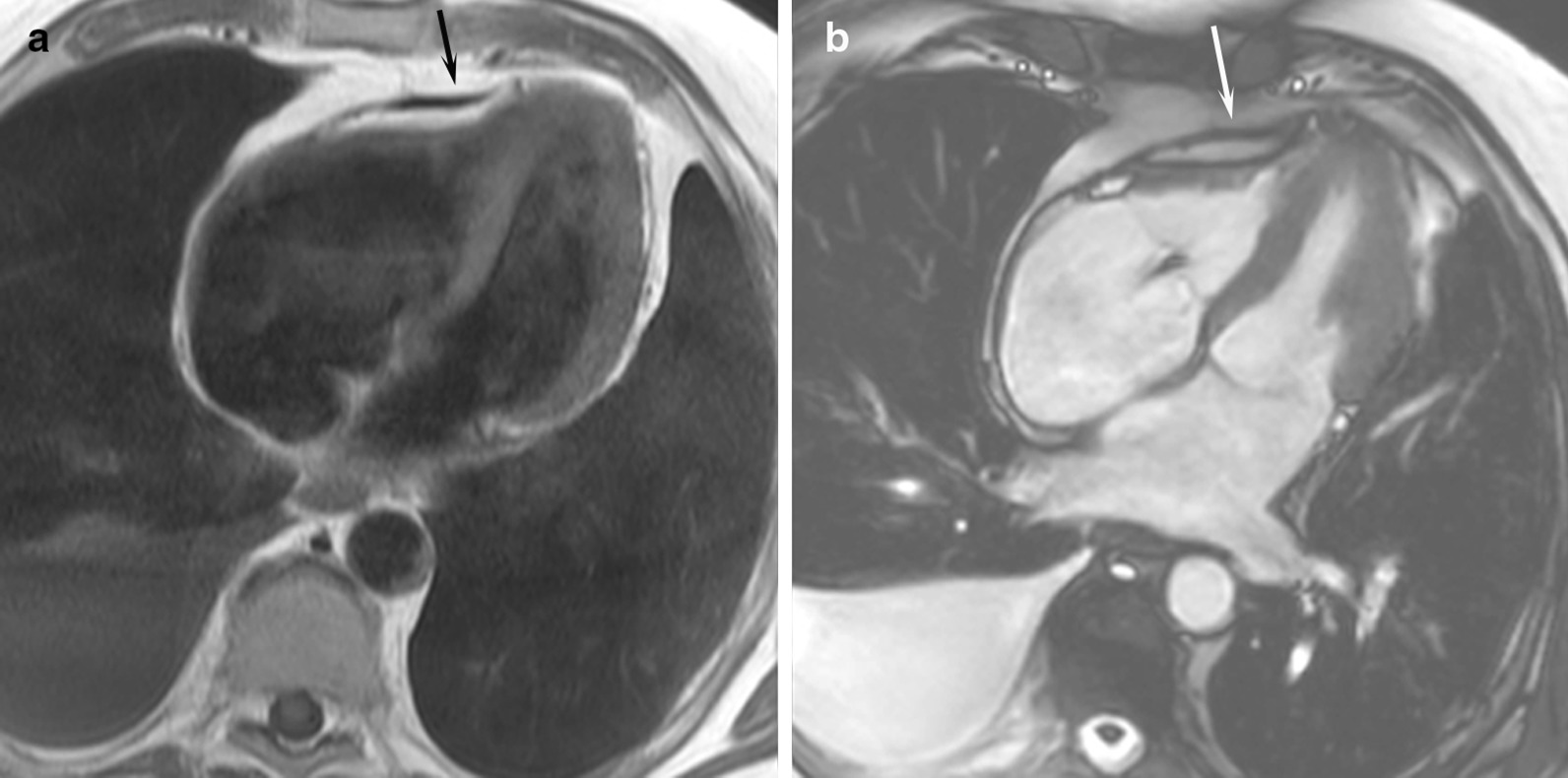
This 72-year-old male was admitted with dyspnea, pleural fluid and cirrhosis. Horizontal long-axis T1-weighted fast spin echo imaging shows (**a**) shows a focally thickened pericardium (black arrow) focally constricting the apical half of the right ventricle (RV). Horizontal long-axis cine imaging (**b**) confirms the constriction of the RV apex by the thick pericardium (white arrow). Presence of a moderate tricuspid regurgitation, bilaterally dilated atria, and right-sided pleural effusion, indirect findings compatible with constrictive pericarditis. The patient underwent successfully a pericardectomy

Patients with pericardial inflammation, even relapsing forms, rarely evolve towards an end-stage constrictive pericarditis [314, 315]. Histologically, the constrictive pericardium consists of collagen-rich fibrous pericardium often with several foci of calcifications. This non-compliant pericardium constricts the heart and may cause diastolic heart failure [323]. Imaging involves description of the pericardium, and on the consequences of the constriction on cardiac morphology and function [314, 315]. Although traditionally regarded as a thick pericardium, thickness as such has a limited role in defining constrictive pericardium. It has been shown that evolution from an inflammatory towards a constrictive pericardium causes a thinning of the abnormally thickened pericardium, likely explaining why at histology a substantial number of patients with constrictive pericarditis presents with mild pericardial thickening (Figs. 10, 11) [324–326]. End-stage constrictive pericardium typically presents with low-signal intensity at T1-weighed spin-echo CMR often with irregular borders. In contrast to pericardial inflammation, T2-weighted spin-echo CMR shows lack of pericardial edema and LGE CMR shows no or limited contrast enhancement [321]. In patients with clinically suspected constrictive pericarditis, the degree of enhancement can be used to predict reversibility and to determine patients who still may benefit of anti-inflammatory treatment [324, 325]. As the pericardium at the atrioventricular sulcus and right side of the heart are most frequently and extensively affected, external compression of the heart at these places is most common. As a consequence, the right atrium and inferior vena cava are dilated, and patients often present pleural effusion [314]. As the morphologic features in constrictive pericarditis may be not impressive, assessment of the hemodynamic consequences is crucial, which can be achieved looking at the ventricular coupling. Constrictive pericarditis patients show pronounced inspiratory flattening and/or inversion of the ventricular septum, while during expiration the opposite phenomenon occurs with increased right-sided septal motion. A simple and elegant way to evaluate ventricular coupling is the use of real-time free breathing bSSFP cine CMR while asking the patient to deep breathe in and out [317]. Also, phase-contrast cine CMR with real-time imaging can be used showing abnormal variation of inflow velocities [318]. Line tagging, with the tag lines perpendicularly positioned on the pericardium may be of interest to assess fibrotic adhesion of the pericardial layers as well as to judge pericardial motion with respect to the underlying myocardial deformation across the cardiac cycle. In patients with constrictive pericarditis, the calcifications are not necessarily confined to the pericardium but may extent into the underlying myocardium, affecting regional and if pronounced also global systolic function [316].

### Congenital abnormalities of the pericardium

Pericardial cyst is the most common pericardial congenital abnormality and is usually an incidental finding in asymptomatic patients [314, 315]. As the location and presentation are typical, the diagnosis is usually straightforward. Pericardial cysts are typically paracardiac in location and present low signal intensity at T1-weighted but high signal at T2-weighted images with well-defined borders. CMR is helpful to differentiate this entity with other cystic structures arising in the chest (bronchogenic, esophageal duplication, thymic). Congenital absence of the pericardium is an extremely rare entity. Protrusion of a portion of the heart through the defect—usually left-sided—causes an abnormal left to posterior location of heart. This observation—in the lack of other explanations for the abnormal positioning—should rise the possibility of a congenital defect [314, 315]. Defining the defect as such is more difficult as the pericardium along the LV is often not well visible due to the lack of surrounding fat. Repositioning the patient in right lateral decubitus may be very helpful to show the dynamic nature of this disease with (near-)normalization of the cardiac configuration/position [327].

## Cardiac masses

Primary tumours of the heart and pericardium, with the exception of atrial myxomas, occur rarely; metastatic tumors to or directly invasive of the heart are far more common [328, 329]. In adults the majority of primary tumors are benign with atrial myxomas being the most common. Other benign tumors include rhabdomyomas, fibromas, papillary fibroelastomas, hemangiomas, lipomas, hamartomas, teratomas and pericardial cysts [328, 329]. The malignant tumors consist of various sarcomas: myxosarcoma, liposarcoma, angiosarcoma, fibrosarcoma, leiomyosarcoma, osteosarcoma, synovial sarcoma, rhabdomyosarcoma, undifferentiated sarcoma, lymphoma, neurofibrosarcoma, and malignant fibrous histiocytoma [328, 329]. Benign tumours in infants and children are mainly teratoma, myxoma, fibroma and rhabdomyoma while malignant tumours include rhabdomyosarcoma, germ cell tumour and fibrosarcoma [330, 331].

Cardiac tumors produce a wide spectrum of symptoms through a number of mechanisms. Their size can obstruct intracardiac blood flow or interfere with valve function. Local invasion can lead to arrhythmias or pericardial effusions with tamponade. Fragments of tumor can embolize, causing systemic deficits when the tumors are on the left side of the heart and pulmonary infarcts on the right side. Finally, the tumors may cause systemic or constitutional symptoms. Some tumors, produce no symptoms and become evident as incidental findings.

**Fig. 12:**
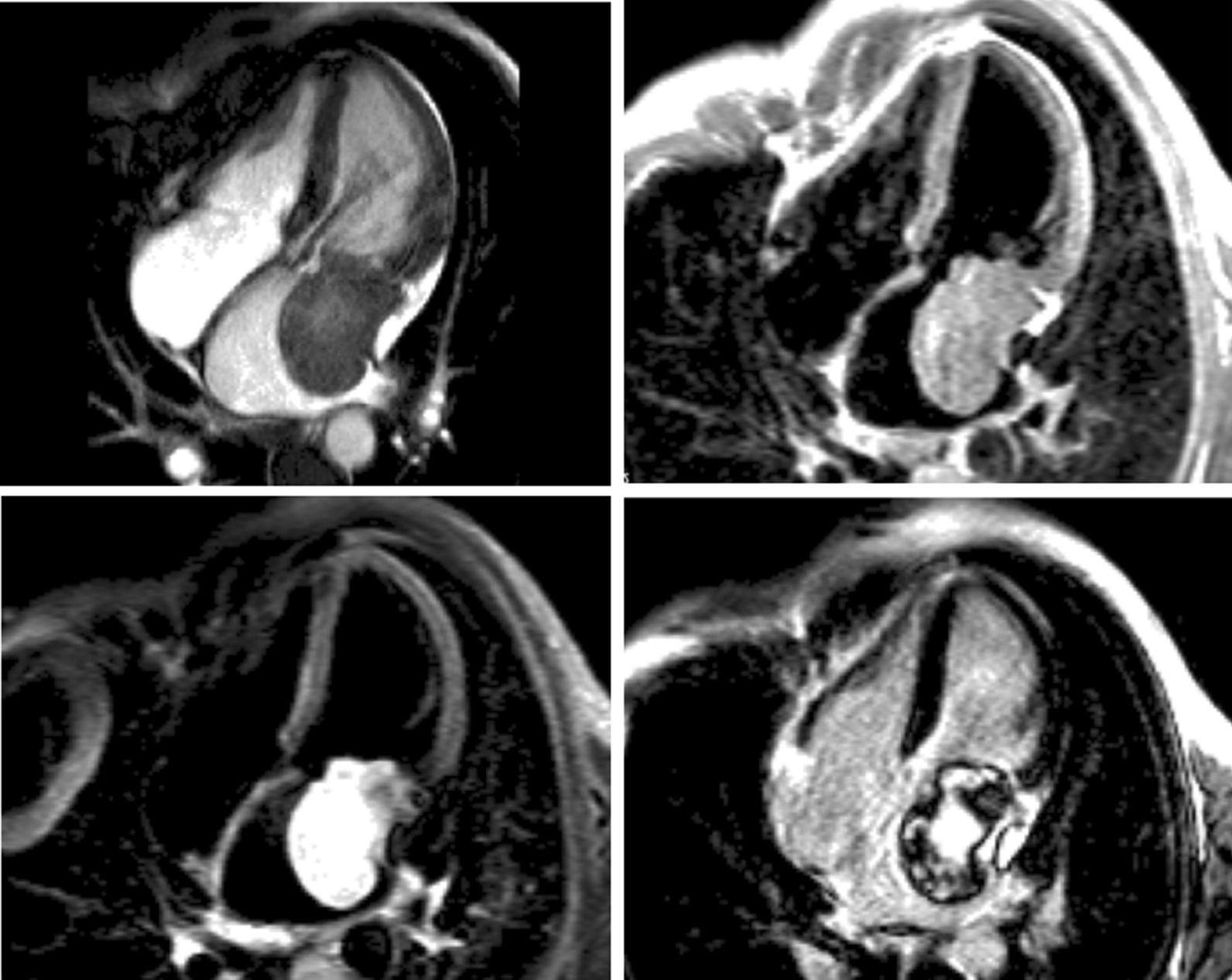
Left atrial sarcoma (undifferentiated) obstructing mitral inflow (upper left: diastolic frame from bSSFP cine). T1W turbo spin-echo image shows the mass isointense relative to the myocardium (upper right) and hyperintense on short tau inversion recovery (STIR) T2 image (lower left). Extensive patchy enhancement within the mass is seen on LGE compatible with necrosis in this setting (lower right)

**Fig. 13:**
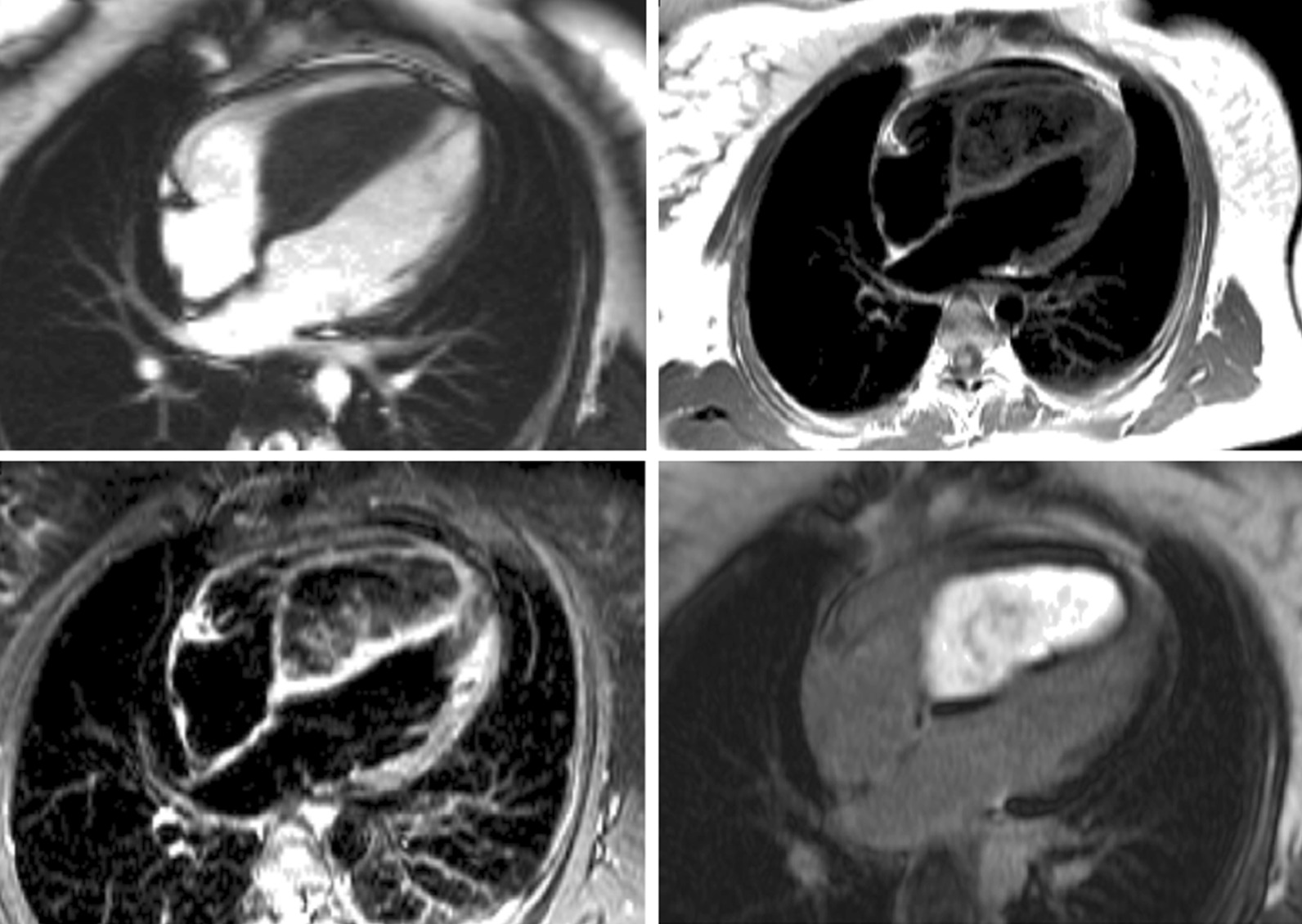
Large right ventricular fibroma attached to the entire ventricular septum. Diastolic frame from bSSFP cine (upper left). T1W fast spin-echo image (upper right) and STIR T2 image (lower right) show hypointense mass with very well defined borders and a thin rim of myocardium. LGE (right lower) shows extensive almost homogenous enhancement of the mass due to high fibrous/collagen content

While TTE remains the first line imaging modality used in patients suspected of an intracardiac tumour or mass, this technique has several limitations and is highly dependent upon availability of good TTE “window” and even then, may not be able to provide a comprehensive answer due to limited field of view and inability to characterize tissue. Positron emission tomography (PET) can also be used to characterize cardiac masses, but it has limited availability and limited spatial resolution and only in combination with the anatomic information of CT has it become a valuable tool for a variety of oncologic indications [332]. CMR has evolved as a reference standard method for the assessment of suspected cardiac tumours and is being increasingly used for confirmation and localization, assessment of size, shape, attachment point and relation to surrounding structures as well as the haemodynamic impact and tissue characterization of cardiac masses that may have been discovered using other types of imaging (Table 6). It is a versatile imaging method that provides 2D or 3D imaging using a variety of pulse sequences for a comprehensive assessment of cardiac tumours and related cardiovascular complications and is helpful in determining prognosis and treatment planning intervention (Figs. 12, 13) [333–335]. Serial CMR studies can be used to evaluate tumour growth, as well as assessment of resection and to monitor recurrence after surgery and to assess regression or progression after chemotherapy or radiotherapy [336, 337]. In addition to this, several CMR features can assist in tumour characterization [333, 334]. The signal intensity of a lesion is dependent on the interaction of the tissue composition and the CMR parameters employed for imaging. The differential diagnosis of a high signal intensity lesion on T1-weighted images, corresponding to a short T1 relaxation time value, includes fatty tumours (lipoma, liposarcoma), recent haemorrhage (due to methaemoglobin breakdown products) and melanoma (due to the effects of melanin). A lesion with low signal intensity on T1-weighted images may represent a cyst filled with low protein fluid, a signal void in a vascular malformation, a calcified lesion or the presence of air. Cysts typically have high signal intensity on T2-weighted images, corresponding to a long T2 relaxation time value, independent of the protein concentration of the fluid. Tumours with high vascularity such as haemangioma also have high signal intensity on T2 weighted images while fibroma exhibits low signal intensity on this sequence. Fat saturation suppress fat signal and can be used to diagnose fatty content definitively or better using fat/water separated images [338].

**Table 6:** Indications for CMR of cardiac masses

Indication	Class
1. Suspected cardiac mass	I
2. Differentiation between benign, malignant and non-tumourous masses	I
3. Guide surgery and/or biopsy if this is deemed appropriate	I
4. Follow-up of benign cardiac tumours that do not require urgent intervention for changes over time	I
5. Evaluation of tumour resection/debulking, monitoring recurrence after surgery and regression or progression after chemotherapy or radiotherapy	I
6. Extra-cardiac extension of cardiac tumours or cardiac extension of tumours originating from surrounding structures	I
7. Impact of cardiac masses on hemodynamics	I

Further differentiation of the tumour can be made with GBCA which can be used in various ways [333–337]. During the first pass of GBCA, vascular tumours show early enhancement and small vessels may be easily identifiable. The first pass enhancement is particularly avid in haemangioma and to a lesser extent in angiosarcoma particularly if there is extensive necrosis and destruction of capillary bed. In the early phase, after injection at 1–2 min, necrotic areas in malignant tumours show as dark areas surrounded by enhancement elsewhere. In the later phase (typically 10 min after injection), benign tumours such fibroma and haemangioma characteristically show strong almost homogenous enhancement while malignant tumours typically show heterogenous contrast enhancement indicating vascularity or GBCA leak age into a necrotic or fibrotic compartment. Such enhancement is usually absent in cystic lesions. Thrombus in the ventricles is well shown by modern CMR sequences, including bSSFP cines, and LGE [339] and for this CMR application may be more sensitive than echocardiography even with echocardiographic contrast [340–342]. CE-CMRA provides additional information about primary or secondary vascular involvement. Intracardiac tumour mobility and its attachment points are best assessed by cine bSSFP while phase-contrast flow mapping is very useful for evaluation of the haemodynamic impact of cardiac tumours. More recently native T1 and T2 mapping have been used as adjunct techniques for tissue characterization of cardiac tumours and masses [343]. Further development of PET/CMR scanners enabling combined high-sensitivity molecular imaging with high soft-tissue contrast and spectroscopic information, may have an important impact on the localization and differentiation of tumors [344].

Finally, it is important to recognize the difference between structures normally present in the heart, which may be mistaken as a cardiac mass, and true cardiac masses. Structures that are normally present in the heart but are sometimes misinterpreted as pathology are [333]:

1. Crista terminalis, seen as a muscular ridge at the entry site of the superior vena cava into the right atrium, demarcating the part of the right atrium that is embryologically derived from the sinus venosus.
2. Eustachian valve, variably present in the right atrium as a remnant structure, after conveying blood toward the foramen ovale during fetal life.
3. “Coumadin ridge” at the confluence of the left upper pulmonary vein and the left atrial appendage which can sometimes be bulbous. This is easily recognized by CMR but it can sometimes be misinterpreted as a thrombus or mass particularly with TTE .
4. Chiari network in the right atrium.
5. The moderator band of the RV that uniquely identifies the anatomical RV;
6. False tendons of the LV.

## Valvular heart disease

CMR has unique capabilities which can greatly enhance the assessment of valvular heart disease. TTE will likely remain the first-line and most common imaging modality for assessing valve disease, and AHA/ACC and ESC guidelines recommend CMR when echocardiographic assessment is unable to provide sufficient information. However, there are many areas where CMR provides ‘added value’ to echocardiographic assessment in valve disease and can be complementary. Further, CMR can provide a comprehensive ‘stand-alone’ assessment in several situations, delivering optimal assessment of patients using a combination of techniques, and for some valve lesions it is the most accurate method of assessment (Table 7).

**Table 7:** Indications for CMR in valvular heart disease

Indication	Class
1. Aortic stenosis	II
2. Identification of sub- and supravalvular stenosis	I
3. Aortic regurgitation	II
4. Ascending aortic flow patterns in aortic stenosis	Inv
5. Mitral stenosis	III
6. Mitral regurgitation	II
7. Pulmonary stenosis	I
8. Pulmonary regurgitation	I
9. Tricuspid stenosis	III
10. Tricuspid regurgitation	II
11. Prosthetic valve disease	II

**Fig. 14:**
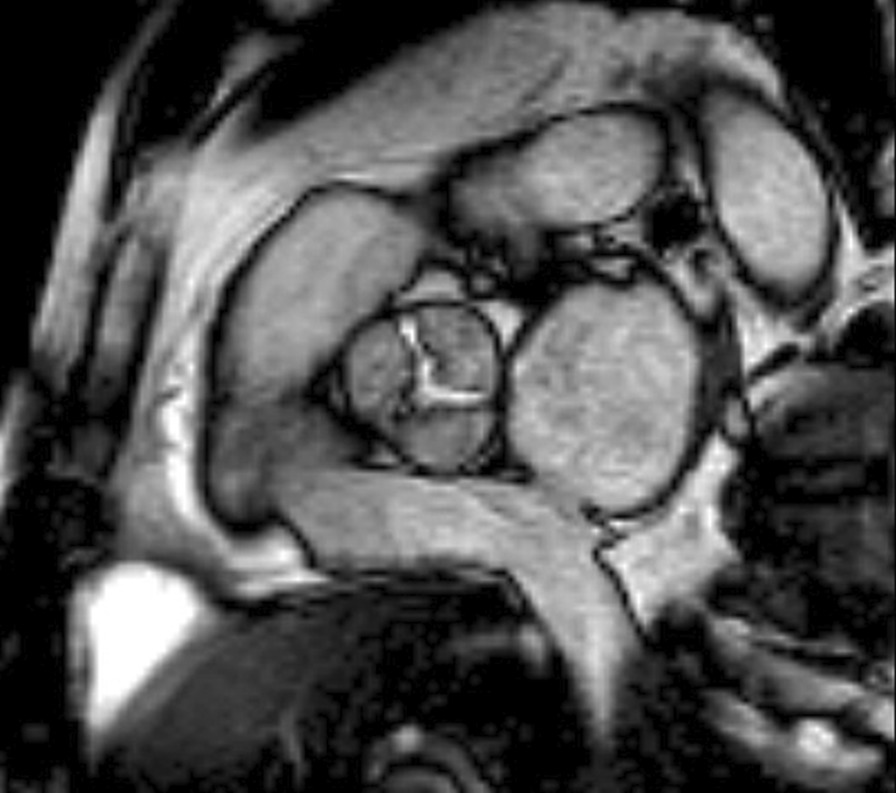
Moderate aortic stenosis (short axis cine bSSFP image through the aortic valve tips in systole)—demonstrating the ease and reliability of direct planimetry to measure the valve area

Cine CMR with bSSFP sequences provides high resolution imaging of valve anatomy and function, particularly utilising the high contrast between the valve leaflets and the blood pool (Fig. 14). Care is required to position the imaging plane perpendicular to the valve leaflets to minimise partial volume effects, but this is readily achieved. Excellent visualisation of the inflow and outflow tracts for both ventricles provide a full assessment of the location and nature of any obstruction. Additional information on the great vessels (aorta and pulmonary trunk) provides important information on the aetiology of aortic and pulmonary valve disease and the surrounding anatomy, to inform clinical management. CMR is especially helpful for areas that can be difficult to view with echocardiography, such as the pulmonary valve/trunk or the ascending aorta and is also feasible in larger patients without compromising image quality or visualisation of the relevant area.

**Fig. 15:**
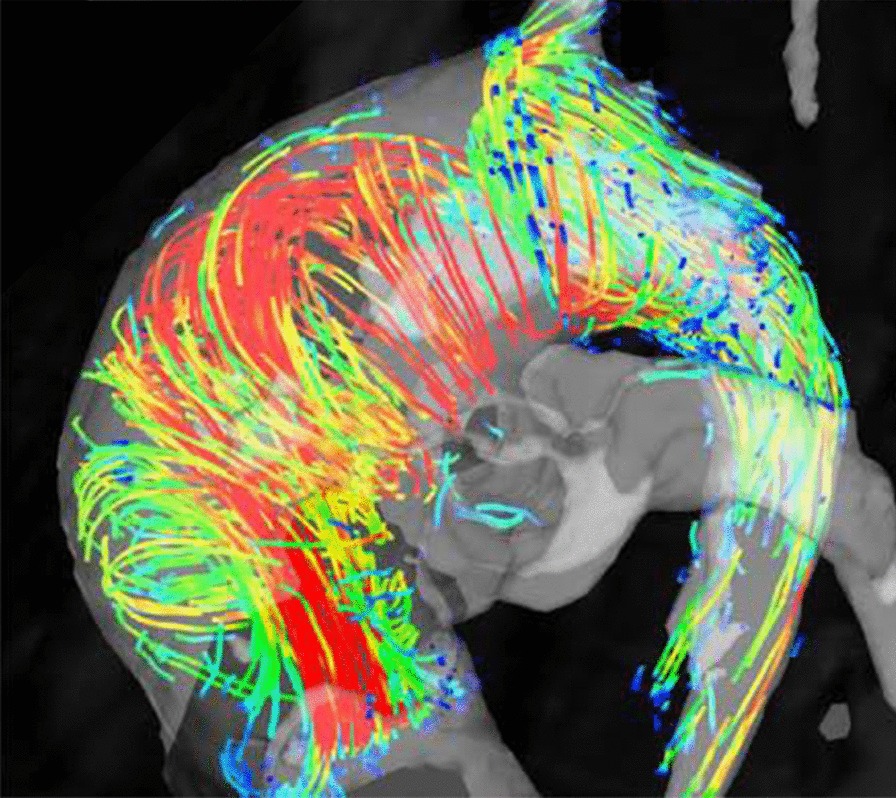
4D flow image demonstrating the extreme helical flow pattern in the ascending aorta of a patient with a bicuspid aortic valve

Phase contrast velocity mapping delivers trans-valvar velocity, similarly to echo, with the advantage of not relying on alignment of the flow jet with the Doppler beam from the external chest wall (Fig. 15). However, the major advantage over all other imaging techniques, and a unique feature of CMR, is the ability of this sequence to quantify flow. This facilitates true quantitation of regurgitant valve lesions, rather than qualitative grading based on arbitrary thresholds.

Finally, accurate and reproducible quantification of LV and RV volumes, mass and function using CMR allows assessment of the effect of the valve lesion(s) on each ventricle and can be monitored over time.

### Regurgitation

Mild regurgitation is usually well assessed with echocardiography and in general does not require CMR assessment. The greatest utility of CMR lies in distinguishing moderate and severe regurgitation—this can be difficult with echocardiography, especially for aortic regurgitation and pulmonary regurgitation with eccentric jets, and also where valve anatomy is non-standard (eg bicuspid valves, cleft mitral valves). Where discrepancy or uncertainty about the degree of regurgitation exists, CMR can usually provide a quantitative answer. Aortic regurgitation and pulmonary regurgitation are straightforward to assess with CMR, using phase contrast velocity mapping in a slice located just distal to the valve, and the regurgitant volume and regurgitant fraction (regurgitant volume/forward volume) can be quantified. It is important to use a reliable correction method for the background flow offset error that can occur, such as the interpolated background flow correction technique. Quantifying mitral regurgitation or tricuspid regurgitation is generally performed using an indirect approach, subtracting aortic flow or pulmonary flow (obtained from flow mapping) from LV or RV stroke volumes, respectively (using whole-heart volumetric cine assessment) [345]. This technique has the advantage of not being affected by regurgitant jets that are multiple, eccentric and/or variable through systole. Newer sequences with valve tracking software and/or 4D flow can be used for direct regurgitation quantification, but these are less well established and available, and their reliability has not been comprehensively assessed.

Quantifying aortic regurgitation with CMR has been shown to predict the future development of symptoms [346], and performed significantly better than TTE. Similar predictive ability has been demonstrated for the quantification of mitral regurgitation with CMR [347, 348], and echocardiography was shown to have a consistent tendency towards over-estimation of the degree of mitral regurgitation [347, 349]. Mitral regurgitation quantification was also strongly associated with LV remodeling after valve repair/replacement [349]. Visualisation of the mitral valve with bSSFP cine imaging provides a similar level of information on anatomy and function as TEE [350], and also provides good assessment of the mitral regurgitant orifice in functional mitral regurgitation [351]. Pulmonary regurgitation is straightforward to quantify and combined with good visualization of the RV outflow tract, CMR is the optimal technique for this lesion (for example in patients with repaired tetralogy of Fallot). In future, evolving techniques such as 4D flow imaging may facilitate accurate direct flow assessment in regurgitant lesions.

### Stenosis

CMR can assess any valve stenosis, although is most commonly used for aortic stenosis and pulmonary stenosis. This is best achieved with a bSSFP cine imaging slice at the valve tips in systole (or diastole for mitral or tricuspid stenosis), which provides clear visualization of the degree of stenosis, and the valve area can be accurately measured from this [352], even with angulated outflow tracts. Although the anatomical valve area is often slightly larger than the valve area assessed by echocardiographic continuity equation, it remains a valuable technique. Velocity mapping adds to this with an assessment of the haemodynamic severity of the stenosis. At higher velocities (> 3-4 m/s), accuracy is reduced due to signal loss from turbulence and phase shift errors from intra-voxel acceleration and dephasing, although ultra-short echo-time velocity mapping sequences may improve accuracy in high velocity jets [353], where these are available. Pulmonary stenosis can be difficult to assess with echocardiography in adults, due to limited acoustic windows and the parallel direction of the RV outflow tract with the sternum, but this is easily assessed with CMR which is the optimal method for assessing pulmonary stenosis. Sub- and supra-valvar aortic and pulmonary stenosis (e.g. sub-aortic membranes) can also be seen easily with CMR bSSFP cine imaging. The presence of myocardial fibrosis on LGE in aortic stenosis has been associated with future events [354] but the clinical utility of this finding requires further study.

### Prosthetic valves

Virtually all prosthetic valves and rings are safe in both 1.5 T and 3 T CMR scanners, and CMR assessment can be valuable where not adequately assessed by echocardiography. Most valves create a signal void artefact, although the degree is variable and depends on the type and amount of metal in the valve or frame. bSSFP or gradient echo cine imaging can visualize prosthetic leaflet opening in selected bioprosthetic valves, as well as demonstrate valve rocking, paravalvar leaks, abscesses and aneurysms where present. Flow quantification beyond the artefact from the valve can quantify forward velocity (for assessment of stenosis) and forward/reverse flow for regurgitation quantification, including the location of the leak. In selected cases, focused regions of interest for flow mapping can quantify the individual components of regurgitation (eg valvar vs paravalvar or each paravalvar leak if > 1 are present) to aid clinical treatment decisions.

## Conclusions

CMR is one of the most powerful diagnostic tools in modern medicine and can provide highly reliable and actionable information for diagnosis and treatment across the spectrum of cardiovascular diseases. The high pace of technical advancements has led to a continuous expansion of the diagnostic capabilities and indications for CMR. In this Society for Cardiovascular Magnetic Resonance Consensus Panel report we provide a contemporary review of indications for CMR. The SCMR intends to update this document frequently with information about new CMR techniques and results of studies and trials that lead to new or altered indications for clinical CMR.

## Data Availability

Not applicable.

## Abbreviations

AC: Arrhythmogenic cardiomyopathy
ACCF: American College of Cardiology Foundation
ACS: Acute coronary syndrome
AHA: American Heart Association
ASTRAL: Angioplasty and Stenting for Renal Artery Lesions trial
bSSFP: Balanced steady state free precession
CAD: Coronary artery disease
CE: Contrast-enhanced
CE-MARC: Cardiovascular magnetic resonance and single-photon emission computed tomography for diagnosis of coronary heart disease
CHD: Congenital heart disease
CKD: Chronic kidney disease
CMP: Cardiomyopathy
CMR: Cardiovascular magnetic resonance
CMRA: Cardiovascular magnetic resonance angiography
CORAL: Cardiovascular Outcomes in Renal Atherosclerotic Lesions trial
COVID-19: Coronavirus disease 19
CT: Computed tomography
CTA: Computed tomography angiography
DCM: Dilated cardiomyopathy
ECG: Electrocardiogram
ECV: Extracellular volume fraction
EF: Ejection fraction
ESC: European Society of Cardiology
FOV: Field of view
GBCA: Gadolinium based contrast agents
HCM: Hypertrophic cardiomyopathy
IMH: Intramural hematoma
ISCHEMIA: International Study of Comparative Health Effectiveness with Medical and Invasive Approaches trial
LGE: Late gadolinium enhancement
LV: Left ventricle/left ventricular
LVEF: Left ventricular ejection fraction
LVNC: Left ventricular noncompaction
LVOT: Left ventricular outflow tract
MI: Myocardial infarction
MINOCA: Myocardial infarction with non-obstructive coronary arteries
MIP: Maximum intensity projection
MO: Microvascular obstruction
MPR: Multiplanar reformation
MRA: Magnetic resonance angiography
MRI: Magnetic resonance imaging
MR-INFORM: MR Perfusion Imaging to Guide Management of Patients With Stable Coronary Artery Disease
NSTEMI: Non-ST elevation myocardial infarction
PET: Positron emission tomography
PIOPED: Prospective investigation of pulmonary embolism diagnosis
PCI: Percutaneous coronary intervention
PWV: Pulse wave velocity
QISS: Quiescent interval single shot
Qp:Qs ratio: Pulmonary to systemic flow ratio
RCT: Randomized clinical trial
RV: Right ventricle/right ventricular
SARS-CoV-2: Severe acute respiratory syndrome corona virus 2
SCMR: Society for Cardiovascular Magnetic Resonance
STEMI: ST-segment elevation myocardial infarction
TE: Echo time
TEE: Transesophageal echocardiography
TEVAR: Thoracic endovascular aortic repair
TGA: Transposition of the great arteries
TI: Inversion time
TR: Repetition time
TSE: Turbo spin echo
TTE: Transthoracic echocardiography
ULP: Ulcer-like projection
VENC: Velocity encoding
VR: Volume rendering
